# Search for lepton-flavour-violating decays of the Higgs and *Z* bosons with the ATLAS detector

**DOI:** 10.1140/epjc/s10052-017-4624-0

**Published:** 2017-02-04

**Authors:** G. Aad, B. Abbott, J. Abdallah, O. Abdinov, B. Abeloos, R. Aben, M. Abolins, O. S. AbouZeid, N. L. Abraham, H. Abramowicz, H. Abreu, R. Abreu, Y. Abulaiti, B. S. Acharya, L. Adamczyk, D. L. Adams, J. Adelman, S. Adomeit, T. Adye, A. A. Affolder, T. Agatonovic-Jovin, J. Agricola, J. A. Aguilar-Saavedra, S. P. Ahlen, F. Ahmadov, G. Aielli, H. Akerstedt, T. P. A. Åkesson, A. V. Akimov, G. L. Alberghi, J. Albert, S. Albrand, M. J. Alconada Verzini, M. Aleksa, I. N. Aleksandrov, C. Alexa, G. Alexander, T. Alexopoulos, M. Alhroob, M. Aliev, G. Alimonti, J. Alison, S. P. Alkire, B. M. M. Allbrooke, B. W. Allen, P. P. Allport, A. Aloisio, A. Alonso, F. Alonso, C. Alpigiani, M. Alstaty, B. Alvarez Gonzalez, D. Álvarez Piqueras, M. G. Alviggi, B. T. Amadio, K. Amako, Y. Amaral Coutinho, C. Amelung, D. Amidei, S. P. Amor Dos Santos, A. Amorim, S. Amoroso, G. Amundsen, C. Anastopoulos, L. S. Ancu, N. Andari, T. Andeen, C. F. Anders, G. Anders, J. K. Anders, K. J. Anderson, A. Andreazza, V. Andrei, S. Angelidakis, I. Angelozzi, P. Anger, A. Angerami, F. Anghinolfi, A. V. Anisenkov, N. Anjos, A. Annovi, M. Antonelli, A. Antonov, J. Antos, F. Anulli, M. Aoki, L. Aperio Bella, G. Arabidze, Y. Arai, J. P. Araque, A. T. H. Arce, F. A. Arduh, J-F. Arguin, S. Argyropoulos, M. Arik, A. J. Armbruster, L. J. Armitage, O. Arnaez, H. Arnold, M. Arratia, O. Arslan, A. Artamonov, G. Artoni, S. Artz, S. Asai, N. Asbah, A. Ashkenazi, B. Åsman, L. Asquith, K. Assamagan, R. Astalos, M. Atkinson, N. B. Atlay, K. Augsten, G. Avolio, B. Axen, M. K. Ayoub, G. Azuelos, M. A. Baak, A. E. Baas, M. J. Baca, H. Bachacou, K. Bachas, M. Backes, M. Backhaus, P. Bagiacchi, P. Bagnaia, Y. Bai, J. T. Baines, O. K. Baker, E. M. Baldin, P. Balek, T. Balestri, F. Balli, W. K. Balunas, E. Banas, Sw. Banerjee, A. A. E. Bannoura, L. Barak, E. L. Barberio, D. Barberis, M. Barbero, T. Barillari, T. Barklow, N. Barlow, S. L. Barnes, B. M. Barnett, R. M. Barnett, Z. Barnovska, A. Baroncelli, G. Barone, A. J. Barr, L. Barranco Navarro, F. Barreiro, J. Barreiro Guimarães da Costa, R. Bartoldus, A. E. Barton, P. Bartos, A. Basalaev, A. Bassalat, R. L. Bates, S. J. Batista, J. R. Batley, M. Battaglia, M. Bauce, F. Bauer, H. S. Bawa, J. B. Beacham, M. D. Beattie, T. Beau, P. H. Beauchemin, P. Bechtle, H. P. Beck, K. Becker, M. Becker, M. Beckingham, C. Becot, A. J. Beddall, A. Beddall, V. A. Bednyakov, M. Bedognetti, C. P. Bee, L. J. Beemster, T. A. Beermann, M. Begel, J. K. Behr, C. Belanger-Champagne, A. S. Bell, G. Bella, L. Bellagamba, A. Bellerive, M. Bellomo, K. Belotskiy, O. Beltramello, N. L. Belyaev, O. Benary, D. Benchekroun, M. Bender, K. Bendtz, N. Benekos, Y. Benhammou, E. Benhar Noccioli, J. Benitez, J. A. Benitez Garcia, D. P. Benjamin, J. R. Bensinger, S. Bentvelsen, L. Beresford, M. Beretta, D. Berge, E. Bergeaas Kuutmann, N. Berger, J. Beringer, S. Berlendis, N. R. Bernard, C. Bernius, F. U. Bernlochner, T. Berry, P. Berta, C. Bertella, G. Bertoli, F. Bertolucci, I. A. Bertram, C. Bertsche, D. Bertsche, G. J. Besjes, O. Bessidskaia Bylund, M. Bessner, N. Besson, C. Betancourt, S. Bethke, A. J. Bevan, W. Bhimji, R. M. Bianchi, L. Bianchini, M. Bianco, O. Biebel, D. Biedermann, R. Bielski, N. V. Biesuz, M. Biglietti, J. Bilbao De Mendizabal, H. Bilokon, M. Bindi, S. Binet, A. Bingul, C. Bini, S. Biondi, D. M. Bjergaard, C. W. Black, J. E. Black, K. M. Black, D. Blackburn, R. E. Blair, J. -B. Blanchard, J. E. Blanco, T. Blazek, I. Bloch, C. Blocker, W. Blum, U. Blumenschein, S. Blunier, G. J. Bobbink, V. S. Bobrovnikov, S. S. Bocchetta, A. Bocci, C. Bock, M. Boehler, D. Boerner, J. A. Bogaerts, D. Bogavac, A. G. Bogdanchikov, C. Bohm, V. Boisvert, P. Bokan, T. Bold, A. S. Boldyrev, M. Bomben, M. Bona, M. Boonekamp, A. Borisov, G. Borissov, J. Bortfeldt, D. Bortoletto, V. Bortolotto, K. Bos, D. Boscherini, M. Bosman, J. D. Bossio Sola, J. Boudreau, J. Bouffard, E. V. Bouhova-Thacker, D. Boumediene, C. Bourdarios, S. K. Boutle, A. Boveia, J. Boyd, I. R. Boyko, J. Bracinik, A. Brandt, G. Brandt, O. Brandt, U. Bratzler, B. Brau, J. E. Brau, H. M. Braun, W. D. Breaden Madden, K. Brendlinger, A. J. Brennan, L. Brenner, R. Brenner, S. Bressler, T. M. Bristow, D. Britton, D. Britzger, F. M. Brochu, I. Brock, R. Brock, G. Brooijmans, T. Brooks, W. K. Brooks, J. Brosamer, E. Brost, J. H Broughton, P. A. Bruckman de Renstrom, D. Bruncko, R. Bruneliere, A. Bruni, G. Bruni, BH Brunt, M. Bruschi, N. Bruscino, P. Bryant, L. Bryngemark, T. Buanes, Q. Buat, P. Buchholz, A. G. Buckley, I. A. Budagov, F. Buehrer, M. K. Bugge, O. Bulekov, D. Bullock, H. Burckhart, S. Burdin, C. D. Burgard, B. Burghgrave, K. Burka, S. Burke, I. Burmeister, E. Busato, D. Büscher, V. Büscher, P. Bussey, J. M. Butler, C. M. Buttar, J. M. Butterworth, P. Butti, W. Buttinger, A. Buzatu, A. R. Buzykaev, S. Cabrera Urbán, D. Caforio, V. M. Cairo, O. Cakir, N. Calace, P. Calafiura, A. Calandri, G. Calderini, P. Calfayan, L. P. Caloba, D. Calvet, S. Calvet, T. P. Calvet, R. Camacho Toro, S. Camarda, P. Camarri, D. Cameron, R. Caminal Armadans, C. Camincher, S. Campana, M. Campanelli, A. Camplani, A. Campoverde, V. Canale, A. Canepa, M. Cano Bret, J. Cantero, R. Cantrill, T. Cao, M. D. M. Capeans Garrido, I. Caprini, M. Caprini, M. Capua, R. Caputo, R. M. Carbone, R. Cardarelli, F. Cardillo, I. Carli, T. Carli, G. Carlino, L. Carminati, S. Caron, E. Carquin, G. D. Carrillo-Montoya, J. R. Carter, J. Carvalho, D. Casadei, M. P. Casado, M. Casolino, D. W. Casper, E. Castaneda-Miranda, A. Castelli, V. Castillo Gimenez, N. F. Castro, A. Catinaccio, J. R. Catmore, A. Cattai, J. Caudron, V. Cavaliere, E. Cavallaro, D. Cavalli, M. Cavalli-Sforza, V. Cavasinni, F. Ceradini, L. Cerda Alberich, B. C. Cerio, A. S. Cerqueira, A. Cerri, L. Cerrito, F. Cerutti, M. Cerv, A. Cervelli, S. A. Cetin, A. Chafaq, D. Chakraborty, S. K. Chan, Y. L. Chan, P. Chang, J. D. Chapman, D. G. Charlton, A. Chatterjee, C. C. Chau, C. A. Chavez Barajas, S. Che, S. Cheatham, A. Chegwidden, S. Chekanov, S. V. Chekulaev, G. A. Chelkov, M. A. Chelstowska, C. Chen, H. Chen, K. Chen, S. Chen, S. Chen, X. Chen, Y. Chen, H. C. Cheng, H. J Cheng, Y. Cheng, A. Cheplakov, E. Cheremushkina, R. Cherkaoui El Moursli, V. Chernyatin, E. Cheu, L. Chevalier, V. Chiarella, G. Chiarelli, G. Chiodini, A. S. Chisholm, A. Chitan, M. V. Chizhov, K. Choi, A. R. Chomont, S. Chouridou, B. K. B. Chow, V. Christodoulou, D. Chromek-Burckhart, J. Chudoba, A. J. Chuinard, J. J. Chwastowski, L. Chytka, G. Ciapetti, A. K. Ciftci, D. Cinca, V. Cindro, I. A. Cioara, A. Ciocio, F. Cirotto, Z. H. Citron, M. Citterio, M. Ciubancan, A. Clark, B. L. Clark, M. R. Clark, P. J. Clark, R. N. Clarke, C. Clement, Y. Coadou, M. Cobal, A. Coccaro, J. Cochran, L. Coffey, L. Colasurdo, B. Cole, S. Cole, A. P. Colijn, J. Collot, T. Colombo, G. Compostella, P. Conde Muiño, E. Coniavitis, S. H. Connell, I. A. Connelly, V. Consorti, S. Constantinescu, C. Conta, G. Conti, F. Conventi, M. Cooke, B. D. Cooper, A. M. Cooper-Sarkar, K. J. R. Cormier, T. Cornelissen, M. Corradi, F. Corriveau, A. Corso-Radu, A. Cortes-Gonzalez, G. Cortiana, G. Costa, M. J. Costa, D. Costanzo, G. Cottin, G. Cowan, B. E. Cox, K. Cranmer, S. J. Crawley, G. Cree, S. Crépé-Renaudin, F. Crescioli, W. A. Cribbs, M. Crispin Ortuzar, M. Cristinziani, V. Croft, G. Crosetti, T. Cuhadar Donszelmann, J. Cummings, M. Curatolo, J. Cúth, C. Cuthbert, H. Czirr, P. Czodrowski, S. D’Auria, M. D’Onofrio, M. J. Da Cunha Sargedas De Sousa, C. Da Via, W. Dabrowski, T. Dado, T. Dai, O. Dale, F. Dallaire, C. Dallapiccola, M. Dam, J. R. Dandoy, N. P. Dang, A. C. Daniells, N. S. Dann, M. Danninger, M. Dano Hoffmann, V. Dao, G. Darbo, S. Darmora, J. Dassoulas, A. Dattagupta, W. Davey, C. David, T. Davidek, M. Davies, P. Davison, E. Dawe, I. Dawson, R. K. Daya-Ishmukhametova, K. De, R. de Asmundis, A. De Benedetti, S. De Castro, S. De Cecco, N. De Groot, P. de Jong, H. De la Torre, F. De Lorenzi, D. De Pedis, A. De Salvo, U. De Sanctis, A. De Santo, J. B. De Vivie De Regie, W. J. Dearnaley, R. Debbe, C. Debenedetti, D. V. Dedovich, I. Deigaard, M. Del Gaudio, J. Del Peso, T. Del Prete, D. Delgove, F. Deliot, C. M. Delitzsch, M. Deliyergiyev, A. Dell’Acqua, L. Dell’Asta, M. Dell’Orso, M. Della Pietra, D. della Volpe, M. Delmastro, P. A. Delsart, C. Deluca, D. A. DeMarco, S. Demers, M. Demichev, A. Demilly, S. P. Denisov, D. Denysiuk, D. Derendarz, J. E. Derkaoui, F. Derue, P. Dervan, K. Desch, C. Deterre, K. Dette, P. O. Deviveiros, A. Dewhurst, S. Dhaliwal, A. Di Ciaccio, L. Di Ciaccio, W. K. Di Clemente, C. Di Donato, A. Di Girolamo, B. Di Girolamo, B. Di Micco, R. Di Nardo, A. Di Simone, R. Di Sipio, D. Di Valentino, C. Diaconu, M. Diamond, F. A. Dias, M. A. Diaz, E. B. Diehl, J. Dietrich, S. Diglio, A. Dimitrievska, J. Dingfelder, P. Dita, S. Dita, F. Dittus, F. Djama, T. Djobava, J. I. Djuvsland, M. A. B. do Vale, D. Dobos, M. Dobre, C. Doglioni, T. Dohmae, J. Dolejsi, Z. Dolezal, B. A. Dolgoshein, M. Donadelli, S. Donati, P. Dondero, J. Donini, J. Dopke, A. Doria, M. T. Dova, A. T. Doyle, E. Drechsler, M. Dris, Y. Du, J. Duarte-Campderros, E. Duchovni, G. Duckeck, O. A. Ducu, D. Duda, A. Dudarev, L. Duflot, L. Duguid, M. Dührssen, M. Dumancic, M. Dunford, H. Duran Yildiz, M. Düren, A. Durglishvili, D. Duschinger, B. Dutta, M. Dyndal, C. Eckardt, K. M. Ecker, R. C. Edgar, N. C. Edwards, T. Eifert, G. Eigen, K. Einsweiler, T. Ekelof, M. El Kacimi, V. Ellajosyula, M. Ellert, S. Elles, F. Ellinghaus, A. A. Elliot, N. Ellis, J. Elmsheuser, M. Elsing, D. Emeliyanov, Y. Enari, O. C. Endner, M. Endo, J. S. Ennis, J. Erdmann, A. Ereditato, G. Ernis, J. Ernst, M. Ernst, S. Errede, E. Ertel, M. Escalier, H. Esch, C. Escobar, B. Esposito, A. I. Etienvre, E. Etzion, H. Evans, A. Ezhilov, F. Fabbri, L. Fabbri, G. Facini, R. M. Fakhrutdinov, S. Falciano, R. J. Falla, J. Faltova, Y. Fang, M. Fanti, A. Farbin, A. Farilla, C. Farina, T. Farooque, S. Farrell, S. M. Farrington, P. Farthouat, F. Fassi, P. Fassnacht, D. Fassouliotis, M. Faucci Giannelli, A. Favareto, W. J. Fawcett, L. Fayard, O. L. Fedin, W. Fedorko, S. Feigl, L. Feligioni, C. Feng, E. J. Feng, H. Feng, A. B. Fenyuk, L. Feremenga, P. Fernandez Martinez, S. Fernandez Perez, J. Ferrando, A. Ferrari, P. Ferrari, R. Ferrari, D. E. Ferreira de Lima, A. Ferrer, D. Ferrere, C. Ferretti, A. Ferretto Parodi, F. Fiedler, A. Filipčič, M. Filipuzzi, F. Filthaut, M. Fincke-Keeler, K. D. Finelli, M. C. N. Fiolhais, L. Fiorini, A. Firan, A. Fischer, C. Fischer, J. Fischer, W. C. Fisher, N. Flaschel, I. Fleck, P. Fleischmann, G. T. Fletcher, R. R. M. Fletcher, T. Flick, A. Floderus, L. R. Flores Castillo, M. J. Flowerdew, G. T. Forcolin, A. Formica, A. Forti, A. G. Foster, D. Fournier, H. Fox, S. Fracchia, P. Francavilla, M. Franchini, D. Francis, L. Franconi, M. Franklin, M. Frate, M. Fraternali, D. Freeborn, S. M. Fressard-Batraneanu, F. Friedrich, D. Froidevaux, J. A. Frost, C. Fukunaga, E. Fullana Torregrosa, T. Fusayasu, J. Fuster, C. Gabaldon, O. Gabizon, A. Gabrielli, A. Gabrielli, G. P. Gach, S. Gadatsch, S. Gadomski, G. Gagliardi, L. G. Gagnon, P. Gagnon, C. Galea, B. Galhardo, E. J. Gallas, B. J. Gallop, P. Gallus, G. Galster, K. K. Gan, J. Gao, Y. Gao, Y. S. Gao, F. M. Garay Walls, C. García, J. E. García Navarro, M. Garcia-Sciveres, R. W. Gardner, N. Garelli, V. Garonne, A. Gascon Bravo, C. Gatti, A. Gaudiello, G. Gaudio, B. Gaur, L. Gauthier, I. L. Gavrilenko, C. Gay, G. Gaycken, E. N. Gazis, Z. Gecse, C. N. P. Gee, Ch. Geich-Gimbel, M. P. Geisler, C. Gemme, M. H. Genest, C. Geng, S. Gentile, S. George, D. Gerbaudo, A. Gershon, S. Ghasemi, H. Ghazlane, M. Ghneimat, B. Giacobbe, S. Giagu, P. Giannetti, B. Gibbard, S. M. Gibson, M. Gignac, M. Gilchriese, T. P. S. Gillam, D. Gillberg, G. Gilles, D. M. Gingrich, N. Giokaris, M. P. Giordani, F. M. Giorgi, F. M. Giorgi, P. F. Giraud, P. Giromini, D. Giugni, F. Giuli, C. Giuliani, M. Giulini, B. K. Gjelsten, S. Gkaitatzis, I. Gkialas, E. L. Gkougkousis, L. K. Gladilin, C. Glasman, J. Glatzer, P. C. F. Glaysher, A. Glazov, M. Goblirsch-Kolb, J. Godlewski, S. Goldfarb, T. Golling, D. Golubkov, A. Gomes, R. Gonçalo, J. Goncalves Pinto Firmino Da Costa, L. Gonella, A. Gongadze, S. González de la Hoz, G. Gonzalez Parra, S. Gonzalez-Sevilla, L. Goossens, P. A. Gorbounov, H. A. Gordon, I. Gorelov, B. Gorini, E. Gorini, A. Gorišek, E. Gornicki, A. T. Goshaw, C. Gössling, M. I. Gostkin, C. R. Goudet, D. Goujdami, A. G. Goussiou, N. Govender, E. Gozani, L. Graber, I. Grabowska-Bold, P. O. J. Gradin, P. Grafström, J. Gramling, E. Gramstad, S. Grancagnolo, V. Gratchev, H. M. Gray, E. Graziani, Z. D. Greenwood, C. Grefe, K. Gregersen, I. M. Gregor, P. Grenier, K. Grevtsov, J. Griffiths, A. A. Grillo, K. Grimm, S. Grinstein, Ph. Gris, J. -F. Grivaz, S. Groh, J. P. Grohs, E. Gross, J. Grosse-Knetter, G. C. Grossi, Z. J. Grout, L. Guan, W. Guan, J. Guenther, F. Guescini, D. Guest, O. Gueta, E. Guido, T. Guillemin, S. Guindon, U. Gul, C. Gumpert, J. Guo, Y. Guo, S. Gupta, G. Gustavino, P. Gutierrez, N. G. Gutierrez Ortiz, C. Gutschow, C. Guyot, C. Gwenlan, C. B. Gwilliam, A. Haas, C. Haber, H. K. Hadavand, N. Haddad, A. Hadef, P. Haefner, S. Hageböck, Z. Hajduk, H. Hakobyan, M. Haleem, J. Haley, G. Halladjian, G. D. Hallewell, K. Hamacher, P. Hamal, K. Hamano, A. Hamilton, G. N. Hamity, P. G. Hamnett, L. Han, K. Hanagaki, K. Hanawa, M. Hance, B. Haney, P. Hanke, R. Hanna, J. B. Hansen, J. D. Hansen, M. C. Hansen, P. H. Hansen, K. Hara, A. S. Hard, T. Harenberg, F. Hariri, S. Harkusha, R. D. Harrington, P. F. Harrison, F. Hartjes, M. Hasegawa, Y. Hasegawa, A. Hasib, S. Hassani, S. Haug, R. Hauser, L. Hauswald, M. Havranek, C. M. Hawkes, R. J. Hawkings, A. D. Hawkins, D. Hayden, C. P. Hays, J. M. Hays, H. S. Hayward, S. J. Haywood, S. J. Head, T. Heck, V. Hedberg, L. Heelan, S. Heim, T. Heim, B. Heinemann, J. J. Heinrich, L. Heinrich, C. Heinz, J. Hejbal, L. Helary, S. Hellman, C. Helsens, J. Henderson, R. C. W. Henderson, Y. Heng, S. Henkelmann, A. M. Henriques Correia, S. Henrot-Versille, G. H. Herbert, Y. Hernández Jiménez, G. Herten, R. Hertenberger, L. Hervas, G. G. Hesketh, N. P. Hessey, J. W. Hetherly, R. Hickling, E. Higón-Rodriguez, E. Hill, J. C. Hill, K. H. Hiller, S. J. Hillier, I. Hinchliffe, E. Hines, R. R. Hinman, M. Hirose, D. Hirschbuehl, J. Hobbs, N. Hod, M. C. Hodgkinson, P. Hodgson, A. Hoecker, M. R. Hoeferkamp, F. Hoenig, M. Hohlfeld, D. Hohn, T. R. Holmes, M. Homann, T. M. Hong, B. H. Hooberman, W. H. Hopkins, Y. Horii, A. J. Horton, J-Y. Hostachy, S. Hou, A. Hoummada, J. Howarth, M. Hrabovsky, I. Hristova, J. Hrivnac, T. Hryn’ova, A. Hrynevich, C. Hsu, P. J. Hsu, S. -C. Hsu, D. Hu, Q. Hu, Y. Huang, Z. Hubacek, F. Hubaut, F. Huegging, T. B. Huffman, E. W. Hughes, G. Hughes, M. Huhtinen, T. A. Hülsing, P. Huo, N. Huseynov, J. Huston, J. Huth, G. Iacobucci, G. Iakovidis, I. Ibragimov, L. Iconomidou-Fayard, E. Ideal, Z. Idrissi, P. Iengo, O. Igonkina, T. Iizawa, Y. Ikegami, M. Ikeno, Y. Ilchenko, D. Iliadis, N. Ilic, T. Ince, G. Introzzi, P. Ioannou, M. Iodice, K. Iordanidou, V. Ippolito, M. Ishino, M. Ishitsuka, R. Ishmukhametov, C. Issever, S. Istin, F. Ito, J. M. Iturbe Ponce, R. Iuppa, W. Iwanski, H. Iwasaki, J. M. Izen, V. Izzo, S. Jabbar, B. Jackson, M. Jackson, P. Jackson, V. Jain, K. B. Jakobi, K. Jakobs, S. Jakobsen, T. Jakoubek, D. O. Jamin, D. K. Jana, E. Jansen, R. Jansky, J. Janssen, M. Janus, G. Jarlskog, N. Javadov, T. Javůrek, F. Jeanneau, L. Jeanty, J. Jejelava, G. -Y. Jeng, D. Jennens, P. Jenni, J. Jentzsch, C. Jeske, S. Jézéquel, H. Ji, J. Jia, H. Jiang, Y. Jiang, S. Jiggins, J. Jimenez Pena, S. Jin, A. Jinaru, O. Jinnouchi, P. Johansson, K. A. Johns, W. J. Johnson, K. Jon-And, G. Jones, R. W. L. Jones, S. Jones, T. J. Jones, J. Jongmanns, P. M. Jorge, J. Jovicevic, X. Ju, A. Juste Rozas, M. K. Köhler, A. Kaczmarska, M. Kado, H. Kagan, M. Kagan, S. J. Kahn, E. Kajomovitz, C. W. Kalderon, A. Kaluza, S. Kama, A. Kamenshchikov, N. Kanaya, S. Kaneti, L. Kanjir, V. A. Kantserov, J. Kanzaki, B. Kaplan, L. S. Kaplan, A. Kapliy, D. Kar, K. Karakostas, A. Karamaoun, N. Karastathis, M. J. Kareem, E. Karentzos, M. Karnevskiy, S. N. Karpov, Z. M. Karpova, K. Karthik, V. Kartvelishvili, A. N. Karyukhin, K. Kasahara, L. Kashif, R. D. Kass, A. Kastanas, Y. Kataoka, C. Kato, A. Katre, J. Katzy, K. Kawagoe, T. Kawamoto, G. Kawamura, S. Kazama, V. F. Kazanin, R. Keeler, R. Kehoe, J. S. Keller, J. J. Kempster, K Kentaro, H. Keoshkerian, O. Kepka, B. P. Kerševan, S. Kersten, R. A. Keyes, F. Khalil-zada, A. Khanov, A. G. Kharlamov, T. J. Khoo, V. Khovanskiy, E. Khramov, J. Khubua, S. Kido, H. Y. Kim, S. H. Kim, Y. K. Kim, N. Kimura, O. M. Kind, B. T. King, M. King, S. B. King, J. Kirk, A. E. Kiryunin, T. Kishimoto, D. Kisielewska, F. Kiss, K. Kiuchi, O. Kivernyk, E. Kladiva, M. H. Klein, M. Klein, U. Klein, K. Kleinknecht, P. Klimek, A. Klimentov, R. Klingenberg, J. A. Klinger, T. Klioutchnikova, E. -E. Kluge, P. Kluit, S. Kluth, J. Knapik, E. Kneringer, E. B. F. G. Knoops, A. Knue, A. Kobayashi, D. Kobayashi, T. Kobayashi, M. Kobel, M. Kocian, P. Kodys, T. Koffas, E. Koffeman, T. Koi, H. Kolanoski, M. Kolb, I. Koletsou, A. A. Komar, Y. Komori, T. Kondo, N. Kondrashova, K. Köneke, A. C. König, T. Kono, R. Konoplich, N. Konstantinidis, R. Kopeliansky, S. Koperny, L. Köpke, A. K. Kopp, K. Korcyl, K. Kordas, A. Korn, A. A. Korol, I. Korolkov, E. V. Korolkova, O. Kortner, S. Kortner, T. Kosek, V. V. Kostyukhin, A. Kotwal, A. Kourkoumeli-Charalampidi, C. Kourkoumelis, V. Kouskoura, A. B. Kowalewska, R. Kowalewski, T. Z. Kowalski, C. Kozakai, W. Kozanecki, A. S. Kozhin, V. A. Kramarenko, G. Kramberger, D. Krasnopevtsev, M. W. Krasny, A. Krasznahorkay, J. K. Kraus, A. Kravchenko, M. Kretz, J. Kretzschmar, K. Kreutzfeldt, P. Krieger, K. Krizka, K. Kroeninger, H. Kroha, J. Kroll, J. Kroseberg, J. Krstic, U. Kruchonak, H. Krüger, N. Krumnack, A. Kruse, M. C. Kruse, M. Kruskal, T. Kubota, H. Kucuk, S. Kuday, J. T. Kuechler, S. Kuehn, A. Kugel, F. Kuger, A. Kuhl, T. Kuhl, V. Kukhtin, R. Kukla, Y. Kulchitsky, S. Kuleshov, M. Kuna, T. Kunigo, A. Kupco, H. Kurashige, Y. A. Kurochkin, V. Kus, E. S. Kuwertz, M. Kuze, J. Kvita, T. Kwan, D. Kyriazopoulos, A. La Rosa, J. L. La Rosa Navarro, L. La Rotonda, C. Lacasta, F. Lacava, J. Lacey, H. Lacker, D. Lacour, V. R. Lacuesta, E. Ladygin, R. Lafaye, B. Laforge, T. Lagouri, S. Lai, S. Lammers, W. Lampl, E. Lançon, U. Landgraf, M. P. J. Landon, V. S. Lang, J. C. Lange, A. J. Lankford, F. Lanni, K. Lantzsch, A. Lanza, S. Laplace, C. Lapoire, J. F. Laporte, T. Lari, F. Lasagni Manghi, M. Lassnig, P. Laurelli, W. Lavrijsen, A. T. Law, P. Laycock, T. Lazovich, M. Lazzaroni, B. Le, O. Le Dortz, E. Le Guirriec, E. P. Le Quilleuc, M. LeBlanc, T. LeCompte, F. Ledroit-Guillon, C. A. Lee, S. C. Lee, L. Lee, G. Lefebvre, M. Lefebvre, F. Legger, C. Leggett, A. Lehan, G. Lehmann Miotto, X. Lei, W. A. Leight, A. Leisos, A. G. Leister, M. A. L. Leite, R. Leitner, D. Lellouch, B. Lemmer, K. J. C. Leney, T. Lenz, B. Lenzi, R. Leone, S. Leone, C. Leonidopoulos, S. Leontsinis, G. Lerner, C. Leroy, A. A. J. Lesage, C. G. Lester, M. Levchenko, J. Levêque, D. Levin, L. J. Levinson, M. Levy, A. M. Leyko, M. Leyton, B. Li, H. Li, H. L. Li, L. Li, L. Li, Q. Li, S. Li, X. Li, Y. Li, Z. Liang, B. Liberti, A. Liblong, P. Lichard, K. Lie, J. Liebal, W. Liebig, A. Limosani, S. C. Lin, T. H. Lin, B. E. Lindquist, E. Lipeles, A. Lipniacka, M. Lisovyi, T. M. Liss, D. Lissauer, A. Lister, A. M. Litke, B. Liu, D. Liu, H. Liu, H. Liu, J. Liu, J. B. Liu, K. Liu, L. Liu, M. Liu, M. Liu, Y. L. Liu, Y. Liu, M. Livan, A. Lleres, J. Llorente Merino, S. L. Lloyd, F. Lo Sterzo, E. Lobodzinska, P. Loch, W. S. Lockman, F. K. Loebinger, A. E. Loevschall-Jensen, K. M. Loew, A. Loginov, T. Lohse, K. Lohwasser, M. Lokajicek, B. A. Long, J. D. Long, R. E. Long, L. Longo, K. A. Looper, L. Lopes, D. Lopez Mateos, B. Lopez Paredes, I. Lopez Paz, A. Lopez Solis, J. Lorenz, N. Lorenzo Martinez, M. Losada, P. J. Lösel, X. Lou, A. Lounis, J. Love, P. A. Love, H. Lu, N. Lu, H. J. Lubatti, C. Luci, A. Lucotte, C. Luedtke, F. Luehring, W. Lukas, L. Luminari, O. Lundberg, B. Lund-Jensen, D. Lynn, R. Lysak, E. Lytken, V. Lyubushkin, H. Ma, L. L. Ma, Y. Ma, G. Maccarrone, A. Macchiolo, C. M. Macdonald, B. Maček, J. Machado Miguens, D. Madaffari, R. Madar, H. J. Maddocks, W. F. Mader, A. Madsen, J. Maeda, S. Maeland, T. Maeno, A. Maevskiy, E. Magradze, J. Mahlstedt, C. Maiani, C. Maidantchik, A. A. Maier, T. Maier, A. Maio, S. Majewski, Y. Makida, N. Makovec, B. Malaescu, Pa. Malecki, V. P. Maleev, F. Malek, U. Mallik, D. Malon, C. Malone, S. Maltezos, S. Malyukov, J. Mamuzic, G. Mancini, B. Mandelli, L. Mandelli, I. Mandić, J. Maneira, L. Manhaes de Andrade Filho, J. Manjarres Ramos, A. Mann, B. Mansoulie, J. D. Mansour, R. Mantifel, M. Mantoani, S. Manzoni, L. Mapelli, G. Marceca, L. March, G. Marchiori, M. Marcisovsky, M. Marjanovic, D. E. Marley, F. Marroquim, S. P. Marsden, Z. Marshall, S. Marti-Garcia, B. Martin, T. A. Martin, V. J. Martin, B. Martin dit Latour, M. Martinez, S. Martin-Haugh, V. S. Martoiu, A. C. Martyniuk, M. Marx, A. Marzin, L. Masetti, T. Mashimo, R. Mashinistov, J. Masik, A. L. Maslennikov, I. Massa, L. Massa, P. Mastrandrea, A. Mastroberardino, T. Masubuchi, P. Mättig, J. Mattmann, J. Maurer, S. J. Maxfield, D. A. Maximov, R. Mazini, S. M. Mazza, N. C. Mc Fadden, G. Mc Goldrick, S. P. Mc Kee, A. McCarn, R. L. McCarthy, T. G. McCarthy, L. I. McClymont, E. F. McDonald, K. W. McFarlane, J. A. Mcfayden, G. Mchedlidze, S. J. McMahon, R. A. McPherson, M. Medinnis, S. Meehan, S. Mehlhase, A. Mehta, K. Meier, C. Meineck, B. Meirose, D. Melini, B. R. Mellado Garcia, M. Melo, F. Meloni, A. Mengarelli, S. Menke, E. Meoni, S. Mergelmeyer, P. Mermod, L. Merola, C. Meroni, F. S. Merritt, A. Messina, J. Metcalfe, A. S. Mete, C. Meyer, C. Meyer, J-P. Meyer, J. Meyer, H. Meyer Zu Theenhausen, F. Miano, R. P. Middleton, S. Miglioranzi, L. Mijović, G. Mikenberg, M. Mikestikova, M. Mikuž, M. Milesi, A. Milic, D. W. Miller, C. Mills, A. Milov, D. A. Milstead, A. A. Minaenko, Y. Minami, I. A. Minashvili, A. I. Mincer, B. Mindur, M. Mineev, Y. Ming, L. M. Mir, K. P. Mistry, T. Mitani, J. Mitrevski, V. A. Mitsou, A. Miucci, P. S. Miyagawa, J. U. Mjörnmark, T. Moa, K. Mochizuki, S. Mohapatra, W. Mohr, S. Molander, R. Moles-Valls, R. Monden, M. C. Mondragon, K. Mönig, J. Monk, E. Monnier, A. Montalbano, J. Montejo Berlingen, F. Monticelli, S. Monzani, R. W. Moore, N. Morange, D. Moreno, M. Moreno Llácer, P. Morettini, D. Mori, T. Mori, M. Morii, M. Morinaga, V. Morisbak, S. Moritz, A. K. Morley, G. Mornacchi, J. D. Morris, S. S. Mortensen, L. Morvaj, M. Mosidze, J. Moss, K. Motohashi, R. Mount, E. Mountricha, S. V. Mouraviev, E. J. W. Moyse, S. Muanza, R. D. Mudd, F. Mueller, J. Mueller, R. S. P. Mueller, T. Mueller, D. Muenstermann, P. Mullen, G. A. Mullier, F. J. Munoz Sanchez, J. A. Murillo Quijada, W. J. Murray, H. Musheghyan, M. Muškinja, A. G. Myagkov, M. Myska, B. P. Nachman, O. Nackenhorst, J. Nadal, K. Nagai, R. Nagai, K. Nagano, Y. Nagasaka, K. Nagata, M. Nagel, E. Nagy, A. M. Nairz, Y. Nakahama, K. Nakamura, T. Nakamura, I. Nakano, H. Namasivayam, R. F. Naranjo Garcia, R. Narayan, D. I. Narrias Villar, I. Naryshkin, T. Naumann, G. Navarro, R. Nayyar, H. A. Neal, P. Yu. Nechaeva, T. J. Neep, P. D. Nef, A. Negri, M. Negrini, S. Nektarijevic, C. Nellist, A. Nelson, S. Nemecek, P. Nemethy, A. A. Nepomuceno, M. Nessi, M. S. Neubauer, M. Neumann, R. M. Neves, P. Nevski, P. R. Newman, D. H. Nguyen, T. Nguyen Manh, R. B. Nickerson, R. Nicolaidou, J. Nielsen, A. Nikiforov, V. Nikolaenko, I. Nikolic-Audit, K. Nikolopoulos, J. K. Nilsen, P. Nilsson, Y. Ninomiya, A. Nisati, R. Nisius, T. Nobe, L. Nodulman, M. Nomachi, I. Nomidis, T. Nooney, S. Norberg, M. Nordberg, N. Norjoharuddeen, O. Novgorodova, S. Nowak, M. Nozaki, L. Nozka, K. Ntekas, E. Nurse, F. Nuti, F. O’grady, D. C. O’Neil, A. A. O’Rourke, V. O’Shea, F. G. Oakham, H. Oberlack, T. Obermann, J. Ocariz, A. Ochi, I. Ochoa, J. P. Ochoa-Ricoux, S. Oda, S. Odaka, H. Ogren, A. Oh, S. H. Oh, C. C. Ohm, H. Ohman, H. Oide, H. Okawa, Y. Okumura, T. Okuyama, A. Olariu, L. F. Oleiro Seabra, S. A. Olivares Pino, D. Oliveira Damazio, A. Olszewski, J. Olszowska, A. Onofre, K. Onogi, P. U. E. Onyisi, M. J. Oreglia, Y. Oren, D. Orestano, N. Orlando, R. S. Orr, B. Osculati, R. Ospanov, G. Otero y Garzon, H. Otono, M. Ouchrif, F. Ould-Saada, A. Ouraou, K. P. Oussoren, Q. Ouyang, M. Owen, R. E. Owen, V. E. Ozcan, N. Ozturk, K. Pachal, A. Pacheco Pages, C. Padilla Aranda, M. Pagáčová, S. Pagan Griso, F. Paige, P. Pais, K. Pajchel, G. Palacino, S. Palestini, M. Palka, D. Pallin, A. Palma, E. St. Panagiotopoulou, C. E. Pandini, J. G. Panduro Vazquez, P. Pani, S. Panitkin, D. Pantea, L. Paolozzi, Th. D. Papadopoulou, K. Papageorgiou, A. Paramonov, D. Paredes Hernandez, A. J. Parker, M. A. Parker, K. A. Parker, F. Parodi, J. A. Parsons, U. Parzefall, V. R. Pascuzzi, E. Pasqualucci, S. Passaggio, F. Pastore, Fr. Pastore, G. Pásztor, S. Pataraia, J. R. Pater, T. Pauly, J. Pearce, B. Pearson, L. E. Pedersen, M. Pedersen, S. Pedraza Lopez, R. Pedro, S. V. Peleganchuk, D. Pelikan, O. Penc, C. Peng, H. Peng, J. Penwell, B. S. Peralva, M. M. Perego, D. V. Perepelitsa, E. Perez Codina, L. Perini, H. Pernegger, S. Perrella, R. Peschke, V. D. Peshekhonov, K. Peters, R. F. Y. Peters, B. A. Petersen, T. C. Petersen, E. Petit, A. Petridis, C. Petridou, P. Petroff, E. Petrolo, M. Petrov, F. Petrucci, N. E. Pettersson, A. Peyaud, R. Pezoa, P. W. Phillips, G. Piacquadio, E. Pianori, A. Picazio, E. Piccaro, M. Piccinini, M. A. Pickering, R. Piegaia, J. E. Pilcher, A. D. Pilkington, A. W. J. Pin, M. Pinamonti, J. L. Pinfold, A. Pingel, S. Pires, H. Pirumov, M. Pitt, L. Plazak, M. -A. Pleier, V. Pleskot, E. Plotnikova, P. Plucinski, D. Pluth, R. Poettgen, L. Poggioli, D. Pohl, G. Polesello, A. Poley, A. Policicchio, R. Polifka, A. Polini, C. S. Pollard, V. Polychronakos, K. Pommès, L. Pontecorvo, B. G. Pope, G. A. Popeneciu, D. S. Popovic, A. Poppleton, S. Pospisil, K. Potamianos, I. N. Potrap, C. J. Potter, C. T. Potter, G. Poulard, J. Poveda, V. Pozdnyakov, M. E. Pozo Astigarraga, P. Pralavorio, A. Pranko, S. Prell, D. Price, L. E. Price, M. Primavera, S. Prince, M. Proissl, K. Prokofiev, F. Prokoshin, S. Protopopescu, J. Proudfoot, M. Przybycien, D. Puddu, D. Puldon, M. Purohit, P. Puzo, J. Qian, G. Qin, Y. Qin, A. Quadt, W. B. Quayle, M. Queitsch-Maitland, D. Quilty, S. Raddum, V. Radeka, V. Radescu, S. K. Radhakrishnan, P. Radloff, P. Rados, F. Ragusa, G. Rahal, J. A. Raine, S. Rajagopalan, M. Rammensee, C. Rangel-Smith, M. G. Ratti, F. Rauscher, S. Rave, T. Ravenscroft, M. Raymond, A. L. Read, N. P. Readioff, D. M. Rebuzzi, A. Redelbach, G. Redlinger, R. Reece, K. Reeves, L. Rehnisch, J. Reichert, H. Reisin, C. Rembser, H. Ren, M. Rescigno, S. Resconi, O. L. Rezanova, P. Reznicek, R. Rezvani, R. Richter, S. Richter, E. Richter-Was, O. Ricken, M. Ridel, P. Rieck, C. J. Riegel, J. Rieger, O. Rifki, M. Rijssenbeek, A. Rimoldi, L. Rinaldi, B. Ristić, E. Ritsch, I. Riu, F. Rizatdinova, E. Rizvi, C. Rizzi, S. H. Robertson, A. Robichaud-Veronneau, D. Robinson, J. E. M. Robinson, A. Robson, C. Roda, Y. Rodina, A. Rodriguez Perez, D. Rodriguez Rodriguez, S. Roe, C. S. Rogan, O. Røhne, A. Romaniouk, M. Romano, S. M. Romano Saez, E. Romero Adam, N. Rompotis, M. Ronzani, L. Roos, E. Ros, S. Rosati, K. Rosbach, P. Rose, O. Rosenthal, N. -A. Rosien, V. Rossetti, E. Rossi, L. P. Rossi, J. H. N. Rosten, R. Rosten, M. Rotaru, I. Roth, J. Rothberg, D. Rousseau, C. R. Royon, A. Rozanov, Y. Rozen, X. Ruan, F. Rubbo, V. I. Rud, M. S. Rudolph, F. Rühr, A. Ruiz-Martinez, Z. Rurikova, N. A. Rusakovich, A. Ruschke, H. L. Russell, J. P. Rutherfoord, N. Ruthmann, Y. F. Ryabov, M. Rybar, G. Rybkin, S. Ryu, A. Ryzhov, G. F. Rzehorz, A. F. Saavedra, G. Sabato, S. Sacerdoti, H. F-W. Sadrozinski, R. Sadykov, F. Safai Tehrani, P. Saha, M. Sahinsoy, M. Saimpert, T. Saito, H. Sakamoto, Y. Sakurai, G. Salamanna, A. Salamon, J. E. Salazar Loyola, D. Salek, P. H. Sales De Bruin, D. Salihagic, A. Salnikov, J. Salt, D. Salvatore, F. Salvatore, A. Salvucci, A. Salzburger, D. Sammel, D. Sampsonidis, A. Sanchez, J. Sánchez, V. Sanchez Martinez, H. Sandaker, R. L. Sandbach, H. G. Sander, M. Sandhoff, C. Sandoval, R. Sandstroem, D. P. C. Sankey, M. Sannino, A. Sansoni, C. Santoni, R. Santonico, H. Santos, I. Santoyo Castillo, K. Sapp, A. Sapronov, J. G. Saraiva, B. Sarrazin, O. Sasaki, Y. Sasaki, K. Sato, G. Sauvage, E. Sauvan, G. Savage, P. Savard, C. Sawyer, L. Sawyer, J. Saxon, C. Sbarra, A. Sbrizzi, T. Scanlon, D. A. Scannicchio, M. Scarcella, V. Scarfone, J. Schaarschmidt, P. Schacht, D. Schaefer, R. Schaefer, J. Schaeffer, S. Schaepe, S. Schaetzel, U. Schäfer, A. C. Schaffer, D. Schaile, R. D. Schamberger, V. Scharf, V. A. Schegelsky, D. Scheirich, M. Schernau, C. Schiavi, C. Schillo, M. Schioppa, S. Schlenker, K. Schmieden, C. Schmitt, S. Schmitt, S. Schmitz, B. Schneider, U. Schnoor, L. Schoeffel, A. Schoening, B. D. Schoenrock, E. Schopf, A. L. S. Schorlemmer, M. Schott, J. Schovancova, S. Schramm, M. Schreyer, N. Schuh, M. J. Schultens, H. -C. Schultz-Coulon, H. Schulz, M. Schumacher, B. A. Schumm, Ph. Schune, C. Schwanenberger, A. Schwartzman, T. A. Schwarz, Ph. Schwegler, H. Schweiger, Ph. Schwemling, R. Schwienhorst, J. Schwindling, T. Schwindt, G. Sciolla, F. Scuri, F. Scutti, J. Searcy, P. Seema, S. C. Seidel, A. Seiden, F. Seifert, J. M. Seixas, G. Sekhniaidze, K. Sekhon, S. J. Sekula, D. M. Seliverstov, N. Semprini-Cesari, C. Serfon, L. Serin, L. Serkin, M. Sessa, R. Seuster, H. Severini, T. Sfiligoj, F. Sforza, A. Sfyrla, E. Shabalina, N. W. Shaikh, L. Y. Shan, R. Shang, J. T. Shank, M. Shapiro, P. B. Shatalov, K. Shaw, S. M. Shaw, A. Shcherbakova, C. Y. Shehu, P. Sherwood, L. Shi, S. Shimizu, C. O. Shimmin, M. Shimojima, M. Shiyakova, A. Shmeleva, D. Shoaleh Saadi, M. J. Shochet, S. Shojaii, S. Shrestha, E. Shulga, M. A. Shupe, P. Sicho, P. E. Sidebo, O. Sidiropoulou, D. Sidorov, A. Sidoti, F. Siegert, Dj. Sijacki, J. Silva, S. B. Silverstein, V. Simak, O. Simard, Lj. Simic, S. Simion, E. Simioni, B. Simmons, D. Simon, M. Simon, P. Sinervo, N. B. Sinev, M. Sioli, G. Siragusa, S. Yu. Sivoklokov, J. Sjölin, T. B. Sjursen, M. B. Skinner, H. P. Skottowe, P. Skubic, M. Slater, T. Slavicek, M. Slawinska, K. Sliwa, R. Slovak, V. Smakhtin, B. H. Smart, L. Smestad, S. Yu. Smirnov, Y. Smirnov, L. N. Smirnova, O. Smirnova, M. N. K. Smith, R. W. Smith, M. Smizanska, K. Smolek, A. A. Snesarev, S. Snyder, R. Sobie, F. Socher, A. Soffer, D. A. Soh, G. Sokhrannyi, C. A. Solans Sanchez, M. Solar, E. Yu. Soldatov, U. Soldevila, A. A. Solodkov, A. Soloshenko, O. V. Solovyanov, V. Solovyev, P. Sommer, H. Son, H. Y. Song, A. Sood, A. Sopczak, V. Sopko, V. Sorin, D. Sosa, C. L. Sotiropoulou, R. Soualah, A. M. Soukharev, D. South, B. C. Sowden, S. Spagnolo, M. Spalla, M. Spangenberg, F. Spanò, D. Sperlich, F. Spettel, R. Spighi, G. Spigo, L. A. Spiller, M. Spousta, R. D. St. Denis, A. Stabile, R. Stamen, S. Stamm, E. Stanecka, R. W. Stanek, C. Stanescu, M. Stanescu-Bellu, M. M. Stanitzki, S. Stapnes, E. A. Starchenko, G. H. Stark, J. Stark, P. Staroba, P. Starovoitov, S. Stärz, R. Staszewski, P. Steinberg, B. Stelzer, H. J. Stelzer, O. Stelzer-Chilton, H. Stenzel, G. A. Stewart, J. A. Stillings, M. C. Stockton, M. Stoebe, G. Stoicea, P. Stolte, S. Stonjek, A. R. Stradling, A. Straessner, M. E. Stramaglia, J. Strandberg, S. Strandberg, A. Strandlie, M. Strauss, P. Strizenec, R. Ströhmer, D. M. Strom, R. Stroynowski, A. Strubig, S. A. Stucci, B. Stugu, N. A. Styles, D. Su, J. Su, R. Subramaniam, S. Suchek, Y. Sugaya, M. Suk, V. V. Sulin, S. Sultansoy, T. Sumida, S. Sun, X. Sun, J. E. Sundermann, K. Suruliz, G. Susinno, M. R. Sutton, S. Suzuki, M. Svatos, M. Swiatlowski, I. Sykora, T. Sykora, D. Ta, C. Taccini, K. Tackmann, J. Taenzer, A. Taffard, R. Tafirout, N. Taiblum, H. Takai, R. Takashima, T. Takeshita, Y. Takubo, M. Talby, A. A. Talyshev, J. Y. C. Tam, K. G. Tan, J. Tanaka, R. Tanaka, S. Tanaka, B. B. Tannenwald, S. Tapia Araya, S. Tapprogge, S. Tarem, G. F. Tartarelli, P. Tas, M. Tasevsky, T. Tashiro, E. Tassi, A. Tavares Delgado, Y. Tayalati, A. C. Taylor, G. N. Taylor, P. T. E. Taylor, W. Taylor, F. A. Teischinger, P. Teixeira-Dias, K. K. Temming, D. Temple, H. Ten Kate, P. K. Teng, J. J. Teoh, F. Tepel, S. Terada, K. Terashi, J. Terron, S. Terzo, M. Testa, R. J. Teuscher, T. Theveneaux-Pelzer, J. P. Thomas, J. Thomas-Wilsker, E. N. Thompson, P. D. Thompson, A. S. Thompson, L. A. Thomsen, E. Thomson, M. Thomson, M. J. Tibbetts, R. E. Ticse Torres, V. O. Tikhomirov, Yu. A. Tikhonov, S. Timoshenko, P. Tipton, S. Tisserant, K. Todome, T. Todorov, S. Todorova-Nova, J. Tojo, S. Tokár, K. Tokushuku, E. Tolley, L. Tomlinson, M. Tomoto, L. Tompkins, K. Toms, B. Tong, E. Torrence, H. Torres, E. Torró Pastor, J. Toth, F. Touchard, D. R. Tovey, T. Trefzger, A. Tricoli, I. M. Trigger, S. Trincaz-Duvoid, M. F. Tripiana, W. Trischuk, B. Trocmé, A. Trofymov, C. Troncon, M. Trottier-McDonald, M. Trovatelli, L. Truong, M. Trzebinski, A. Trzupek, J. C-L. Tseng, P. V. Tsiareshka, G. Tsipolitis, N. Tsirintanis, S. Tsiskaridze, V. Tsiskaridze, E. G. Tskhadadze, K. M. Tsui, I. I. Tsukerman, V. Tsulaia, S. Tsuno, D. Tsybychev, A. Tudorache, V. Tudorache, A. N. Tuna, S. A. Tupputi, S. Turchikhin, D. Turecek, D. Turgeman, R. Turra, A. J. Turvey, P. M. Tuts, M. Tyndel, G. Ucchielli, I. Ueda, R. Ueno, M. Ughetto, F. Ukegawa, G. Unal, A. Undrus, G. Unel, F. C. Ungaro, Y. Unno, C. Unverdorben, J. Urban, P. Urquijo, P. Urrejola, G. Usai, A. Usanova, L. Vacavant, V. Vacek, B. Vachon, C. Valderanis, E. Valdes Santurio, N. Valencic, S. Valentinetti, A. Valero, L. Valery, S. Valkar, S. Vallecorsa, J. A. Valls Ferrer, W. Van Den Wollenberg, P. C. Van Der Deijl, R. van der Geer, H. van der Graaf, N. van Eldik, P. van Gemmeren, J. Van Nieuwkoop, I. van Vulpen, M. C. van Woerden, M. Vanadia, W. Vandelli, R. Vanguri, A. Vaniachine, P. Vankov, G. Vardanyan, R. Vari, E. W. Varnes, T. Varol, D. Varouchas, A. Vartapetian, K. E. Varvell, J. G. Vasquez, F. Vazeille, T. Vazquez Schroeder, J. Veatch, L. M. Veloce, F. Veloso, S. Veneziano, A. Ventura, M. Venturi, N. Venturi, A. Venturini, V. Vercesi, M. Verducci, W. Verkerke, J. C. Vermeulen, A. Vest, M. C. Vetterli, O. Viazlo, I. Vichou, T. Vickey, O. E. Vickey Boeriu, G. H. A. Viehhauser, S. Viel, L. Vigani, R. Vigne, M. Villa, M. Villaplana Perez, E. Vilucchi, M. G. Vincter, V. B. Vinogradov, C. Vittori, I. Vivarelli, S. Vlachos, M. Vlasak, M. Vogel, P. Vokac, G. Volpi, M. Volpi, H. von der Schmitt, E. von Toerne, V. Vorobel, K. Vorobev, M. Vos, R. Voss, J. H. Vossebeld, N. Vranjes, M. Vranjes Milosavljevic, V. Vrba, M. Vreeswijk, R. Vuillermet, I. Vukotic, Z. Vykydal, P. Wagner, W. Wagner, H. Wahlberg, S. Wahrmund, J. Wakabayashi, J. Walder, R. Walker, W. Walkowiak, V. Wallangen, C. Wang, C. Wang, F. Wang, H. Wang, H. Wang, J. Wang, J. Wang, K. Wang, R. Wang, S. M. Wang, T. Wang, T. Wang, X. Wang, C. Wanotayaroj, A. Warburton, C. P. Ward, D. R. Wardrope, A. Washbrook, P. M. Watkins, A. T. Watson, M. F. Watson, G. Watts, S. Watts, B. M. Waugh, S. Webb, M. S. Weber, S. W. Weber, J. S. Webster, A. R. Weidberg, B. Weinert, J. Weingarten, C. Weiser, H. Weits, P. S. Wells, T. Wenaus, T. Wengler, S. Wenig, N. Wermes, M. Werner, P. Werner, M. Wessels, J. Wetter, K. Whalen, N. L. Whallon, A. M. Wharton, A. White, M. J. White, R. White, S. White, D. Whiteson, F. J. Wickens, W. Wiedenmann, M. Wielers, P. Wienemann, C. Wiglesworth, L. A. M. Wiik-Fuchs, A. Wildauer, F. Wilk, H. G. Wilkens, H. H. Williams, S. Williams, C. Willis, S. Willocq, J. A. Wilson, I. Wingerter-Seez, F. Winklmeier, O. J. Winston, B. T. Winter, M. Wittgen, J. Wittkowski, S. J. Wollstadt, M. W. Wolter, H. Wolters, B. K. Wosiek, J. Wotschack, M. J. Woudstra, K. W. Wozniak, M. Wu, M. Wu, S. L. Wu, X. Wu, Y. Wu, T. R. Wyatt, B. M. Wynne, S. Xella, D. Xu, L. Xu, B. Yabsley, S. Yacoob, R. Yakabe, D. Yamaguchi, Y. Yamaguchi, A. Yamamoto, S. Yamamoto, T. Yamanaka, K. Yamauchi, Y. Yamazaki, Z. Yan, H. Yang, H. Yang, Y. Yang, Z. Yang, W-M. Yao, Y. C. Yap, Y. Yasu, E. Yatsenko, K. H. Yau Wong, J. Ye, S. Ye, I. Yeletskikh, A. L. Yen, E. Yildirim, K. Yorita, R. Yoshida, K. Yoshihara, C. Young, C. J. S. Young, S. Youssef, D. R. Yu, J. Yu, J. M. Yu, J. Yu, L. Yuan, S. P. Y. Yuen, I. Yusuff, B. Zabinski, R. Zaidan, A. M. Zaitsev, N. Zakharchuk, J. Zalieckas, A. Zaman, S. Zambito, L. Zanello, D. Zanzi, C. Zeitnitz, M. Zeman, A. Zemla, J. C. Zeng, Q. Zeng, K. Zengel, O. Zenin, T. Ženiš, D. Zerwas, D. Zhang, F. Zhang, G. Zhang, H. Zhang, J. Zhang, L. Zhang, R. Zhang, R. Zhang, X. Zhang, Z. Zhang, X. Zhao, Y. Zhao, Z. Zhao, A. Zhemchugov, J. Zhong, B. Zhou, C. Zhou, L. Zhou, L. Zhou, M. Zhou, N. Zhou, C. G. Zhu, H. Zhu, J. Zhu, Y. Zhu, X. Zhuang, K. Zhukov, A. Zibell, D. Zieminska, N. I. Zimine, C. Zimmermann, S. Zimmermann, Z. Zinonos, M. Zinser, M. Ziolkowski, L. Živković, G. Zobernig, A. Zoccoli, M. zur Nedden, G. Zurzolo, L. Zwalinski, ATLAS Collaboration

**Affiliations:** 1 Department of Physics, University of Adelaide, Adelaide, SA Australia; 2 Physics Department, SUNY Albany, Albany, NY USA; 3 Department of Physics, University of Alberta, Edmonton, AB Canada; 4 Department of Physics, Ankara University, Ankara, Turkey; 5 Istanbul Aydin University, Istanbul, Turkey; 6 Division of Physics, TOBB University of Economics and Technology, Ankara, Turkey; 7 LAPP, CNRS/IN2P3 and Université Savoie Mont Blanc, Annecy-le-Vieux, France; 8 High Energy Physics Division, Argonne National Laboratory, Argonne, IL USA; 9 Department of Physics, University of Arizona, Tucson, AZ USA; 10 Department of Physics, The University of Texas at Arlington, Arlington, TX USA; 11 Physics Department, University of Athens, Athens, Greece; 12 Physics Department, National Technical University of Athens, Zografou, Greece; 13 Department of Physics, The University of Texas at Austin, Austin, TX USA; 14 Institute of Physics, Azerbaijan Academy of Sciences, Baku, Azerbaijan; 15 Institut de Física d’Altes Energies (IFAE), The Barcelona Institute of Science and Technology, Barcelona, Spain; 16 Institute of Physics, University of Belgrade, Belgrade, Serbia; 17 Department for Physics and Technology, University of Bergen, Bergen, Norway; 18 Physics Division, Lawrence Berkeley National Laboratory and University of California, Berkeley, CA USA; 19 Department of Physics, Humboldt University, Berlin, Germany; 20 Albert Einstein Center for Fundamental Physics and Laboratory for High Energy Physics, University of Bern, Bern, Switzerland; 21 School of Physics and Astronomy, University of Birmingham, Birmingham, UK; 22 Department of Physics, Bogazici University, Istanbul, Turkey; 23 Department of Physics Engineering, Gaziantep University, Gaziantep, Turkey; 24 Istanbul Bilgi University, Faculty of Engineering and Natural Sciences, Istanbul, Turkey; 25 Bahcesehir University, Faculty of Engineering and Natural Sciences, Istanbul, Turkey; 26 Centro de Investigaciones, Universidad Antonio Narino, Bogota, Colombia; 27 INFN Sezione di Bologna, Bologna, Italy; 28 Dipartimento di Fisica e Astronomia, Università di Bologna, Bologna, Italy; 29 Physikalisches Institut, University of Bonn, Bonn, Germany; 30 Department of Physics, Boston University, Boston, MA USA; 31 Department of Physics, Brandeis University, Waltham, MA USA; 32 Universidade Federal do Rio De Janeiro COPPE/EE/IF, Rio de Janeiro, Brazil; 33 Electrical Circuits Department, Federal University of Juiz de Fora (UFJF), Juiz de Fora, Brazil; 34 Federal University of Sao Joao del Rei (UFSJ), Sao Joao del Rei, Brazil; 35 Instituto de Fisica, Universidade de Sao Paulo, Sao Paulo, Brazil; 36 Physics Department, Brookhaven National Laboratory, Upton, NY USA; 37 Transilvania University of Brasov, Brasov, Romania; 38 National Institute of Physics and Nuclear Engineering, Bucharest, Romania; 39 Physics Department, National Institute for Research and Development of Isotopic and Molecular Technologies, Cluj Napoca, Romania; 40 University Politehnica Bucharest, Bucharest, Romania; 41 West University in Timisoara, Timisoara, Romania; 42 Departamento de Física, Universidad de Buenos Aires, Buenos Aires, Argentina; 43 Cavendish Laboratory, University of Cambridge, Cambridge, UK; 44 Department of Physics, Carleton University, Ottawa, ON Canada; 45 CERN, Geneva, Switzerland; 46 Enrico Fermi Institute, University of Chicago, Chicago, IL USA; 47 Departamento de Física, Pontificia Universidad Católica de Chile, Santiago, Chile; 48 Departamento de Física, Universidad Técnica Federico Santa María, Valparaiso, Chile; 49 Institute of High Energy Physics, Chinese Academy of Sciences, Beijing, China; 50 Department of Modern Physics, University of Science and Technology of China, Anhui, China; 51 Department of Physics, Nanjing University, Jiangsu, China; 52 School of Physics, Shandong University, Shandong, China; 53 Department of Physics and Astronomy, Shanghai Key Laboratory for Particle Physics and Cosmology, Shanghai Jiao Tong University (also affiliated with PKU-CHEP), Shanghai, China; 54 Physics Department, Tsinghua University, Beijing, 100084 China; 55 Laboratoire de Physique Corpusculaire, Clermont Université and Université Blaise Pascal and CNRS/IN2P3, Clermont-Ferrand, France; 56 Nevis Laboratory, Columbia University, Irvington, NY USA; 57 Niels Bohr Institute, University of Copenhagen, Copenhagen, Denmark; 58 INFN Gruppo Collegato di Cosenza, Laboratori Nazionali di Frascati, Frascati, Italy; 59 Dipartimento di Fisica, Università della Calabria, Rende, Italy; 60 Faculty of Physics and Applied Computer Science, AGH University of Science and Technology, Krakow, Poland; 61 Marian Smoluchowski Institute of Physics, Jagiellonian University, Kraków, Poland; 62 Institute of Nuclear Physics, Polish Academy of Sciences, Kraków, Poland; 63 Physics Department, Southern Methodist University, Dallas, TX USA; 64 Physics Department, University of Texas at Dallas, Richardson, TX USA; 65 DESY, Hamburg and Zeuthen, Germany; 66 Institut für Experimentelle Physik IV, Technische Universität Dortmund, Dortmund, Germany; 67 Institut für Kern-und Teilchenphysik, Technische Universität Dresden, Dresden, Germany; 68 Department of Physics, Duke University, Durham, NC USA; 69 SUPA-School of Physics and Astronomy, University of Edinburgh, Edinburgh, UK; 70 INFN Laboratori Nazionali di Frascati, Frascati, Italy; 71 Fakultät für Mathematik und Physik, Albert-Ludwigs-Universität, Freiburg, Germany; 72 Section de Physique, Université de Genève, Geneva, Switzerland; 73 INFN Sezione di Genova, Genoa, Italy; 74 Dipartimento di Fisica, Università di Genova, Genoa, Italy; 75 E. Andronikashvili Institute of Physics, Iv. Javakhishvili Tbilisi State University, Tbilisi, Georgia; 76 High Energy Physics Institute, Tbilisi State University, Tbilisi, Georgia; 77 II Physikalisches Institut, Justus-Liebig-Universität Giessen, Giessen, Germany; 78 SUPA-School of Physics and Astronomy, University of Glasgow, Glasgow, UK; 79 II Physikalisches Institut, Georg-August-Universität, Göttingen, Germany; 80 Laboratoire de Physique Subatomique et de Cosmologie, Université Grenoble-Alpes, CNRS/IN2P3, Grenoble, France; 81 Department of Physics, Hampton University, Hampton, VA USA; 82 Laboratory for Particle Physics and Cosmology, Harvard University, Cambridge, MA USA; 83 Kirchhoff-Institut für Physik, Ruprecht-Karls-Universität Heidelberg, Heidelberg, Germany; 84 Physikalisches Institut, Ruprecht-Karls-Universität Heidelberg, Heidelberg, Germany; 85 ZITI Institut für technische Informatik, Ruprecht-Karls-Universität Heidelberg, Mannheim, Germany; 86 Faculty of Applied Information Science, Hiroshima Institute of Technology, Hiroshima, Japan; 87 Department of Physics, The Chinese University of Hong Kong, Shatin, NT Hong Kong; 88 Department of Physics, The University of Hong Kong, Hong Kong, China; 89 Department of Physics, The Hong Kong University of Science and Technology, Clear Water Bay, Kowloon, Hong Kong, China; 90 Department of Physics, Indiana University, Bloomington, IN USA; 91 Institut für Astro- und Teilchenphysik, Leopold-Franzens-Universität, Innsbruck, Austria; 92 University of Iowa, Iowa City, IA USA; 93 Department of Physics and Astronomy, Iowa State University, Ames, IA USA; 94 Joint Institute for Nuclear Research, JINR Dubna, Dubna, Russia; 95 KEK, High Energy Accelerator Research Organization, Tsukuba, Japan; 96 Graduate School of Science, Kobe University, Kobe, Japan; 97 Faculty of Science, Kyoto University, Kyoto, Japan; 98 Kyoto University of Education, Kyoto, Japan; 99 Department of Physics, Kyushu University, Fukuoka, Japan; 100 Instituto de Física La Plata, Universidad Nacional de La Plata and CONICET, La Plata, Argentina; 101 Physics Department, Lancaster University, Lancaster, UK; 102 INFN Sezione di Lecce, Lecce, Italy; 103 Dipartimento di Matematica e Fisica, Università del Salento, Lecce, Italy; 104 Oliver Lodge Laboratory, University of Liverpool, Liverpool, UK; 105 Department of Physics, Jožef Stefan Institute, University of Ljubljana, Ljubljana, Slovenia; 106 School of Physics and Astronomy, Queen Mary University of London, London, UK; 107 Department of Physics, Royal Holloway University of London, Surrey, UK; 108 Department of Physics and Astronomy, University College London, London, UK; 109 Louisiana Tech University, Ruston, LA USA; 110 Laboratoire de Physique Nucléaire et de Hautes Energies, UPMC and Université Paris-Diderot and CNRS/IN2P3, Paris, France; 111 Fysiska institutionen, Lunds universitet, Lund, Sweden; 112 Departamento de Fisica Teorica C-15, Universidad Autonoma de Madrid, Madrid, Spain; 113 Institut für Physik, Universität Mainz, Mainz, Germany; 114 School of Physics and Astronomy, University of Manchester, Manchester, UK; 115 CPPM, Aix-Marseille Université and CNRS/IN2P3, Marseille, France; 116 Department of Physics, University of Massachusetts, Amherst, MA USA; 117 Department of Physics, McGill University, Montreal, QC Canada; 118 School of Physics, University of Melbourne, Melbourne, VIC Australia; 119 Department of Physics, The University of Michigan, Ann Arbor, MI USA; 120 Department of Physics and Astronomy, Michigan State University, East Lansing, MI USA; 121 INFN Sezione di Milano, Milan, Italy; 122 Dipartimento di Fisica, Università di Milano, Milan, Italy; 123 B.I. Stepanov Institute of Physics, National Academy of Sciences of Belarus, Minsk, Republic of Belarus; 124 National Scientific and Educational Centre for Particle and High Energy Physics, Minsk, Republic of Belarus; 125 Group of Particle Physics, University of Montreal, Montreal, QC Canada; 126 P.N. Lebedev Physical Institute of the Russian Academy of Sciences, Moscow, Russia; 127 Institute for Theoretical and Experimental Physics (ITEP), Moscow, Russia; 128 National Research Nuclear University MEPhI, Moscow, Russia; 129 D.V. Skobeltsyn Institute of Nuclear Physics, M.V. Lomonosov Moscow State University, Moscow, Russia; 130 Fakultät für Physik, Ludwig-Maximilians-Universität München, Munich, Germany; 131 Max-Planck-Institut für Physik (Werner-Heisenberg-Institut), Munich, Germany; 132 Nagasaki Institute of Applied Science, Nagasaki, Japan; 133 Graduate School of Science and Kobayashi-Maskawa Institute, Nagoya University, Nagoya, Japan; 134 INFN Sezione di Napoli, Naples, Italy; 135 Dipartimento di Fisica, Università di Napoli, Naples, Italy; 136 Department of Physics and Astronomy, University of New Mexico, Albuquerque, NM USA; 137 Institute for Mathematics, Astrophysics and Particle Physics, Radboud University Nijmegen/Nikhef, Nijmegen, The Netherlands; 138 Nikhef National Institute for Subatomic Physics and University of Amsterdam, Amsterdam, The Netherlands; 139 Department of Physics, Northern Illinois University, DeKalb, IL USA; 140 Budker Institute of Nuclear Physics, SB RAS, Novosibirsk, Russia; 141 Department of Physics, New York University, New York, NY USA; 142 Ohio State University, Columbus, OH USA; 143 Faculty of Science, Okayama University, Okayama, Japan; 144 Homer L. Dodge Department of Physics and Astronomy, University of Oklahoma, Norman, OK USA; 145 Department of Physics, Oklahoma State University, Stillwater, OK USA; 146 Palacký University, RCPTM, Olomouc, Czech Republic; 147 Center for High Energy Physics, University of Oregon, Eugene, OR USA; 148 LAL, Univ. Paris-Sud, CNRS/IN2P3, Université Paris-Saclay, Orsay, France; 149 Graduate School of Science, Osaka University, Osaka, Japan; 150 Department of Physics, University of Oslo, Oslo, Norway; 151 Department of Physics, Oxford University, Oxford, UK; 152 INFN Sezione di Pavia, Pavia, Italy; 153 Dipartimento di Fisica, Università di Pavia, Pavia, Italy; 154 Department of Physics, University of Pennsylvania, Philadelphia, PA USA; 155 National Research Centre “Kurchatov Institute” B.P.Konstantinov Petersburg Nuclear Physics Institute, St. Petersburg, Russia; 156 INFN Sezione di Pisa, Pisa, Italy; 157 Dipartimento di Fisica E. Fermi, Università di Pisa, Pisa, Italy; 158 Department of Physics and Astronomy, University of Pittsburgh, Pittsburgh, PA USA; 159 Laboratório de Instrumentação e Física Experimental de Partículas-LIP, Lisbon, Portugal; 160 Faculdade de Ciências, Universidade de Lisboa, Lisbon, Portugal; 161 Department of Physics, University of Coimbra, Coimbra, Portugal; 162 Centro de Física Nuclear da Universidade de Lisboa, Lisbon, Portugal; 163 Departamento de Fisica, Universidade do Minho, Braga, Portugal; 164 Departamento de Fisica Teorica y del Cosmos and CAFPE, Universidad de Granada, Granada, Spain; 165 Dep Fisica and CEFITEC of Faculdade de Ciencias e Tecnologia, Universidade Nova de Lisboa, Caparica, Portugal; 166 Institute of Physics, Academy of Sciences of the Czech Republic, Prague, Czech Republic; 167 Czech Technical University in Prague, Prague, Czech Republic; 168 Faculty of Mathematics and Physics, Charles University in Prague, Prague, Czech Republic; 169 State Research Center Institute for High Energy Physics (Protvino), NRC KI, Russia; 170 Particle Physics Department, Rutherford Appleton Laboratory, Didcot, UK; 171 INFN Sezione di Roma, Rome, Italy; 172 Dipartimento di Fisica, Sapienza Università di Roma, Rome, Italy; 173 INFN Sezione di Roma Tor Vergata, Rome, Italy; 174 Dipartimento di Fisica, Università di Roma Tor Vergata, Rome, Italy; 175 INFN Sezione di Roma Tre, Rome, Italy; 176 Dipartimento di Matematica e Fisica, Università Roma Tre, Rome, Italy; 177 Faculté des Sciences Ain Chock, Réseau Universitaire de Physique des Hautes Energies-Université Hassan II, Casablanca, Morocco; 178 Centre National de l’Energie des Sciences Techniques Nucleaires, Rabat, Morocco; 179 Faculté des Sciences Semlalia, Université Cadi Ayyad, LPHEA-Marrakech, Marrakech, Morocco; 180 Faculté des Sciences, Université Mohamed Premier and LPTPM, Oujda, Morocco; 181 Faculté des Sciences, Université Mohammed V, Rabat, Morocco; 182 DSM/IRFU (Institut de Recherches sur les Lois Fondamentales de l’Univers), CEA Saclay (Commissariat à l’Energie Atomique et aux Energies Alternatives), Gif-sur-Yvette, France; 183 Santa Cruz Institute for Particle Physics, University of California Santa Cruz, Santa Cruz, CA USA; 184 Department of Physics, University of Washington, Seattle, WA USA; 185 Department of Physics and Astronomy, University of Sheffield, Sheffield, UK; 186 Department of Physics, Shinshu University, Nagano, Japan; 187 Fachbereich Physik, Universität Siegen, Siegen, Germany; 188 Department of Physics, Simon Fraser University, Burnaby, BC Canada; 189 SLAC National Accelerator Laboratory, Stanford, CA USA; 190 Faculty of Mathematics, Physics and Informatics, Comenius University, Bratislava, Slovak Republic; 191 Department of Subnuclear Physics, Institute of Experimental Physics of the Slovak Academy of Sciences, Kosice, Slovak Republic; 192 Department of Physics, University of Cape Town, Cape Town, South Africa; 193 Department of Physics, University of Johannesburg, Johannesburg, South Africa; 194 School of Physics, University of the Witwatersrand, Johannesburg, South Africa; 195 Department of Physics, Stockholm University, Stockholm, Sweden; 196 The Oskar Klein Centre, Stockholm, Sweden; 197 Physics Department, Royal Institute of Technology, Stockholm, Sweden; 198 Departments of Physics and Astronomy and Chemistry, Stony Brook University, Stony Brook, NY USA; 199 Department of Physics and Astronomy, University of Sussex, Brighton, UK; 200 School of Physics, University of Sydney, Sydney, NSW Australia; 201 Institute of Physics, Academia Sinica, Taipei, Taiwan; 202 Department of Physics, Technion: Israel Institute of Technology, Haifa, Israel; 203 Raymond and Beverly Sackler School of Physics and Astronomy, Tel Aviv University, Tel Aviv, Israel; 204 Department of Physics, Aristotle University of Thessaloniki, Thessaloniki, Greece; 205 International Center for Elementary Particle Physics and Department of Physics, The University of Tokyo, Tokyo, Japan; 206 Graduate School of Science and Technology, Tokyo Metropolitan University, Tokyo, Japan; 207 Department of Physics, Tokyo Institute of Technology, Tokyo, Japan; 208 Department of Physics, University of Toronto, Toronto, ON Canada; 209 TRIUMF, Vancouver, BC Canada; 210 Department of Physics and Astronomy, York University, Toronto, ON Canada; 211 Faculty of Pure and Applied Sciences, and Center for Integrated Research in Fundamental Science and Engineering, University of Tsukuba, Tsukuba, Japan; 212 Department of Physics and Astronomy, Tufts University, Medford, MA USA; 213 Department of Physics and Astronomy, University of California Irvine, Irvine, CA USA; 214 INFN Gruppo Collegato di Udine, Sezione di Trieste, Udine, Italy; 215 ICTP, Trieste, Italy; 216 Dipartimento di Chimica Fisica e Ambiente, Università di Udine, Udine, Italy; 217 Department of Physics and Astronomy, University of Uppsala, Uppsala, Sweden; 218 Department of Physics, University of Illinois, Urbana, IL USA; 219 Instituto de Fisica Corpuscular (IFIC) and Departamento de Fisica Atomica, Molecular y Nuclear and Departamento de Ingeniería Electrónica and Instituto de Microelectrónica de Barcelona (IMB-CNM), University of Valencia and CSIC, Valencia, Spain; 220 Department of Physics, University of British Columbia, Vancouver, BC Canada; 221 Department of Physics and Astronomy, University of Victoria, Victoria, BC Canada; 222 Department of Physics, University of Warwick, Coventry, UK; 223 Waseda University, Tokyo, Japan; 224 Department of Particle Physics, The Weizmann Institute of Science, Rehovot, Israel; 225 Department of Physics, University of Wisconsin, Madison, WI USA; 226 Fakultät für Physik und Astronomie, Julius-Maximilians-Universität, Würzburg, Germany; 227 Fakultät für Mathematik und Naturwissenschaften, Fachgruppe Physik, Bergische Universität Wuppertal, Wuppertal, Germany; 228 Department of Physics, Yale University, New Haven, CT USA; 229 Yerevan Physics Institute, Yerevan, Armenia; 230 Centre de Calcul de l’Institut National de Physique Nucléaire et de Physique des Particules (IN2P3), Villeurbanne, France; 231 CERN, 1211 Geneva 23, Switzerland

## Abstract

Direct searches for lepton flavour violation in decays of the Higgs and *Z* bosons with the ATLAS detector at the LHC are presented. The following three decays are considered: $H\rightarrow e\tau$, $H\rightarrow \mu \tau$, and $Z\rightarrow \mu \tau$. The searches are based on the data sample of proton–proton collisions collected by the ATLAS detector corresponding to an integrated luminosity of 20.3 $\mathrm{fb}^{-1}$ at a centre-of-mass energy of $\sqrt{s}=8$ TeV. No significant excess is observed, and upper limits on the lepton-flavour-violating branching ratios are set at the 95$\%$ confidence level: Br$(H\rightarrow e\tau )<1.04\%$, Br$(H\rightarrow \mu \tau )<1.43\%$, and Br$(Z\rightarrow \mu \tau )<1.69\times 10^{-5}$.

## Introduction

One of the main goals of the Large Hadron Collider (LHC) physics programme at CERN is to discover physics beyond the Standard Model (SM). A possible sign would be the observation of lepton flavour violation (LFV) that could be realised in decays of the Higgs boson or of the *Z* boson to pairs of leptons with different flavours.

Lepton-flavour-violating decays of the Higgs boson can occur naturally in models with more than one Higgs doublet [1–4], composite Higgs models [5, 6], models with flavour symmetries [7], Randall–Sundrum models [8] and many others [9–16]. LFV *Z* boson decays are predicted in models with heavy neutrinos [17], extended gauge models [18] and supersymmetry [19].

The most stringent bounds on the LFV decays of the Higgs and *Z* bosons other than $H\rightarrow {}\mu {}e$ are derived from direct searches [20]. The CMS Collaboration has performed the first direct search for LFV $H\rightarrow \mu \tau$ decays [21] and reported a small excess (2.4 standard deviations) of data over the predicted background. Their results give a 1.51$\%$ upper limit on Br($H\rightarrow \mu \tau$) at the 95$\%$ confidence level (CL). The ATLAS Collaboration has also performed a search [22] for the LFV $H\rightarrow \mu \tau$ decays in the final state with one muon and one hadronically decaying $\tau$-lepton, $\tau _{\mathrm {had}} {}$, and reported a 1.85$\%$ upper limit on Br($H\rightarrow \mu \tau$) at the 95$\%$ CL. The most stringent indirect constraint on $H\rightarrow e\mu$ decays is derived from the results of searches for $\mu \rightarrow e\gamma$ decays [23], and a bound of Br($H\rightarrow e\mu$) < O($10^{-8}$) is obtained [24, 25]. The bound on $\mu \rightarrow e\gamma$ decays suggests that the presence of a $H\rightarrow \mu \tau$ signal would exclude the presence of a $H\rightarrow e\tau$ signal, and vice versa, at an experimentally observable level at the LHC [25]. It is also important to note that a relatively large Br($H\rightarrow \mu \tau$) can be achieved without any particular tuning of the effective couplings, while a large Br($H\rightarrow e\tau$) is possible only at the cost of some fine-tuning of the corresponding couplings [25]. Upper bounds on the LFV $Z\rightarrow e\mu$, $Z\rightarrow \mu \tau$ and $Z\rightarrow e\tau$ decays were set by the LEP experiments [26, 27]: Br$(Z\rightarrow e\mu )<1.7\times 10^{-6}$, Br$(Z\rightarrow e\tau )<9.8\times 10^{-6}$, and Br$(Z\rightarrow \mu \tau )<1.2\times 10^{-5}$ at the 95$\%$ CL. The ATLAS experiment set the most stringent upper bound on the LFV $Z\rightarrow e\mu$ decays [28]: Br$(Z\rightarrow e\mu )<7.5 \times 10^{-7}$ at 95$\%$ CL.

This paper describes three new searches for LFV decays of the Higgs and *Z* bosons. The first study is a search for $H\rightarrow e\tau$ decays in the final state with one electron and one hadronically decaying $\tau$-lepton, $\tau _{\mathrm {had}}$. The second analysis is a simultaneous search for the LFV $H\rightarrow e\tau$ and $H\rightarrow \mu \tau$ decays in the final state with a leptonically decaying $\tau$-lepton, $\tau _{\mathrm {lep}} {}$. A combination of results of the earlier ATLAS search for the LFV $H\rightarrow \mu \tau _{\mathrm {had}}$ decays [22] and the two searches described in this paper is also presented. The third study constitutes the first ATLAS search for LFV decays of the *Z* boson with hadronic $\tau$-lepton decays in the channel $Z\rightarrow \mu {} \tau _{\mathrm {had}} {}$. The search for LFV decays in the $\tau _{\mathrm {lep}} {}$ analysis is based on the novel method introduced in Ref. [29]; the searches in the $\tau _{\mathrm {had}} {}$ analyses are based on the techniques developed for the SM $H\rightarrow \tau _{\mathrm {lep}} {} \tau _{\mathrm {had}} {}$ search. All three searches are based on the data sample of *pp* collisions collected at a centre-of-mass energy of $\sqrt{s}=8$ TeV and corresponding to an integrated luminosity of 20.3 $\mathrm{fb}^{-1}$. Given the overlap between the analysis techniques used in the $H\rightarrow {}e \tau _{\mathrm {had}}$ search and in the $Z\rightarrow \mu {} \tau _{\mathrm {had}} {}$ search, from here on they are referred to as the $\tau _{\mathrm {had}}$ channels; the $H\rightarrow {}\ell \tau _{\mathrm {lep}}$ search is referred to as the $\tau _{\mathrm {lep}}$ channel, where $\ell {}=e, \mu {}$.

## The ATLAS detector and object reconstruction

The ATLAS detector^1^ is described in detail in Ref. [30]. ATLAS consists of an inner tracking detector (ID) covering the range $|\eta |<2.5$, surrounded by a superconducting solenoid providing a 2 T axial magnetic field, a high-granularity electromagnetic ($|\eta |<3.2$) calorimeter, a hadronic calorimeter ($|\eta |<4.9$), and a muon spectrometer (MS) ($|\eta |<2.7$) with a toroidal magnetic field.

The signatures of LFV searches reported here are characterised by the presence of an energetic lepton originating directly from the boson decay and carrying roughly half of its energy, and the hadronic or leptonic decay products of a $\tau$-lepton. The data in the $\tau _{\mathrm {had}}$ channels were collected with single-lepton triggers: a single-muon trigger with the threshold of $p_{\text {T}} =24$ GeV and a single-electron trigger with the threshold $E_{\text {T}} =24$ GeV. The data in the $\tau _{\mathrm {lep}}$ channel were collected using asymmetric electron-muon triggers with $(p_{\text {T}} ^{\mu },E_{\text {T}} ^{e})>(18, 8)$ GeV and $(E_{\text {T}} ^{e},p_{\text {T}} ^{\mu })>(14,8)$ GeV thresholds. The $p_{\text {T}}$ and $E_{\text {T}}$ requirements on the objects in the presented analyses are at least 2 GeV higher than the trigger requirements.

A brief description of the object definitions is provided below. The primary vertex is chosen as the proton–proton collision vertex candidate with the highest sum of the squared transverse momenta of all associated tracks [31].

Muon candidates are reconstructed using an algorithm that combines information from the ID and the MS [32]. Muon quality criteria such as inner-detector hit requirements are applied to achieve a precise measurement of the muon momentum and to reduce the misidentification rate. Muons are required to have $p_{\text {T}} >10$ GeV and to be within $|\eta |<2.5$. The distance between the *z*-position of the point of closest approach of the muon inner-detector track to the beam-line and the *z*-coordinate of the primary vertex is required to be less than 1 cm. In the $\tau _{\mathrm {lep}}$ channel, there is an additional cut on the transverse impact parameter significance, defined as the transverse impact parameter divided by its uncertainty: $|d_0|/\sigma _{d_0}<3$. These requirements reduce the contamination due to cosmic-ray muons and beam-induced backgrounds. Typical reconstruction and identification efficiencies for muons meeting these selection criteria are above 95% [32].

Electron candidates are reconstructed from energy clusters in the electromagnetic calorimeters matched to tracks in the ID. They are required to have transverse energy $E_{\text {T}} {}>15(12)~\mathrm{GeV}{}$ in the $\tau _{\mathrm {had}}$ ($\tau _{\mathrm {lep}}$) channel, to be within the pseudorapidity range $|\eta |<2.47$, and to satisfy the *medium* shower shape and track selection criteria defined in Ref. [33]. Candidates found in the transition region between the barrel and end-cap calorimeters ($1.37<|\eta |<1.52$) are not considered in the $\tau _{\mathrm {had}}$ channel. Typical reconstruction and identification efficiencies for electrons satisfying these selection criteria range between 80 and 90%, depending on $E_{\text {T}}$ and $\eta$.

Exactly one lepton (electron or muon) satisfying the above identification requirements is allowed in the $\tau _{\mathrm {had}}$ channels. In the $\tau _{\mathrm {lep}}$ channel, only events with exactly one identified muon and one identified electron are retained. All lepton (electron or muon) candidates must be matched to the corresponding trigger objects and satisfy additional isolation criteria, based on tracking and calorimeter information, in order to suppress the background from misidentified jets or from semileptonic decays of charm and bottom hadrons. The calorimeter isolation variable $I(E_{\text {T}},\Delta R)$ is defined as the sum of the total transverse energy in the calorimeter in a cone of size $\Delta {}R$ around the electron cluster or the muon track, divided by the $E_{\text {T}}$ of the electron cluster or the $p_{\text {T}}$ of the muon, respectively. The track-based isolation $I(p_{\text {T}},\Delta R)$ is defined as the scalar sum of the transverse momenta of tracks within a cone of size $\Delta R$ around the electron or muon track, divided by the $E_{\text {T}}$ of the electron cluster or the muon $p_{\text {T}}$, respectively. The contribution due to the lepton itself is not included in either sum. The isolation requirements used in the $\tau _{\mathrm {had}}$ and $\tau _{\mathrm {lep}}$ channels, optimised to reduce the contamination from non-prompt leptons, are listed in Table 1.

**Table 1:** Summary of isolation requirements applied for the selection of isolated electrons and muons. The isolation variables are defined in the text

	$\tau _{\mathrm {lep}}$ channels	$\tau _{\mathrm {had}}$ channels
Electrons	$I(E_{\text {T}},0.3)<0.13$	$I(E_{\text {T}},0.2)<0.06$
	$I(p_{\text {T}},0.3)<0.07$	$I(p_{\text {T}},0.4)<0.06$
Muons	$I(E_{\text {T}},0.3)<0.14$	$I(E_{\text {T}},0.2)<0.06$
	$I(p_{\text {T}},0.3)<0.06$	$I(p_{\text {T}},0.4)<0.06$

Hadronically decaying $\tau$-leptons are identified by means of a multivariate analysis technique [34] based on boosted decision trees, which exploits information about ID tracks and clusters in the electromagnetic and hadronic calorimeters. The $\tau _{\mathrm {had}}$ candidates are required to have $+1$ or $-1$ net charge in units of electron charge, and must be 1- or 3-track (1- or 3-prong) candidates. Events with exactly one $\tau _{\mathrm {had}}$ candidate satisfying the *medium* identification criteria [34] with $p_{\text {T}} >20$ GeV and $|\eta |<2.47$ are considered in the $\tau _{\mathrm {had}}$ channels. In the $\tau _{\mathrm {lep}}$ channel, events with identified $\tau _{\mathrm {had}}$ candidates are rejected to avoid overlap between $H\rightarrow {}\ell \tau _{\mathrm {had}}$ and $H\rightarrow {}\ell \tau _{\mathrm {lep}}$. The identification efficiency for $\tau _{\mathrm {had}}$ candidates satisfying these requirements is (55–60)%. Dedicated criteria [34] to separate $\tau _{\mathrm {had}}$ candidates from misidentified electrons are also applied, with a selection efficiency for true $\tau _{\mathrm {had}}$ decays (that pass the $\tau _{\mathrm {had}}$ identification requirements described above) of 95%. To reduce the contamination due to backgrounds where a muon mimics a $\tau _{\mathrm {had}}$ signature, events in which an identified muon with $p_{\text {T}} (\mu )>4$ GeV overlaps with an identified $\tau _{\mathrm {had}}$ are rejected [35]. The probability to misidentify a jet with $p_{\text {T}} >20$ GeV as a $\tau _{\mathrm {had}}$ candidate is typically (1–2)% [34].

Jets are reconstructed using the anti-$k_{t}$ jet clustering algorithm [36] with a radius parameter $R=0.4$, taking the deposited energy in clusters of calorimeter cells as inputs. Fully calibrated jets [37] are required to be reconstructed in the range $|\eta |<4.5$ and to have $p_{\text {T}} >30$ GeV. To suppress jets from multiple proton–proton collisions in the same or nearby beam bunch crossings, tracking information is used for central jets with $|\eta |<2.4$ and $p_{\text {T}} <50$ GeV. In the $\tau _{\mathrm {lep}}$ channel, these central jets are required to have at least one track originating from the primary vertex. In the $\tau _{\mathrm {had}}$ channel, tracks originating from the primary vertex must contribute more than half of the jet $p_{\text {T}}$ when summing the scalar $p_{\text {T}}$ of all tracks in the jet; jets with no associated tracks are retained.

In the pseudorapidity range $|\eta |<2.5$, jets containing *b*-hadrons (*b*-jets) are selected using a tagging algorithm [38]. These jets are required to have $p_{\text {T}} {}>30~\mathrm{GeV}{}$ in the $\tau _{\mathrm {had}}$ channel, and $p_{\text {T}} {}>20~\mathrm{GeV}{}$ in the $\tau _{\mathrm {lep}}$ channel. Two different working points with $\sim$70 and $\sim$80$\%$ *b*-tagging efficiencies for *b*-jets in simulated $t\bar{t}$ events are used in the $\tau _{\mathrm {had}}$ and $\tau _{\mathrm {lep}}$ channels, respectively. The corresponding light-flavour jet misidentification probability is (0.1–1)%, depending on the $p_{\text {T}}$ and $\eta$ of the jet. Only a very small fraction of signal events have *b*-jets, therefore events with identified *b*-jets are vetoed in the selection of signal events.

Some objects might be reconstructed as more than one candidate. Overlapping candidates, separeted by $\Delta R < 0.2$, are resolved by discarding one object and selecting the other one in the following order of priority (from highest to lowest): muons, electrons, $\tau _{\mathrm {had}}$, and jet candidates [35].

The missing transverse momentum (with magnitude $E_{\text {T}}^{\text {miss}}$) is reconstructed using the energy deposits in calorimeter cells calibrated according to the reconstructed physics objects (*e*, $\gamma$, $\tau _{\mathrm {had}}$, jets and $\mu$) with which they are associated [39]. In the $\tau _{\mathrm {had}}$ channels, the energy from calorimeter cells not associated with any physics object is included in the $E_{\text {T}}^{\text {miss}}$ calculation. It is scaled by the scalar sum of $p_{\text {T}}$ of tracks which originate from the primary vertex but are not associated with any objects divided by the scalar sum of $p_{\text {T}}$ of all tracks in the event which are not associated with objects. The scaling procedure achieves a more accurate reconstruction of $E_{\text {T}}^{\text {miss}}$ under high pile-up conditions.

## Signal and background samples

The LFV signal is estimated from simulation. The major Higgs boson production processes (gluon fusion *ggH*, vector-boson fusion VBF, and associated production *WH* / *ZH*) are considered in the reported searches for LFV $H\rightarrow e\tau$ and $H\rightarrow \mu \tau$ decays. In the $\tau _{\mathrm {lep}}$ channel, all backgrounds are estimated from data. In the $\tau _{\mathrm {had}}$ channels, the $Z/\gamma ^{*}\rightarrow \tau \tau$ and multi-jet backgrounds are estimated from data, while the other remaining backgrounds are estimated from simulation, as described below.

The largely irreducible $Z/\gamma ^{*}\rightarrow \tau \tau$ background is modelled by $Z/\gamma ^{*}\rightarrow \mu \mu$ data events, where the muon tracks and associated energy deposits in the calorimeters are replaced by the corresponding simulated signatures of the final-state particles of the $\tau$-lepton decay. In this approach, essential features such as the modelling of the kinematics of the produced boson, the modelling of the hadronic activity of the event (jets and underlying event) as well as contributions from pile-up are taken from data. Therefore, the dependence on the simulation is minimised and only the $\tau$-lepton decays and the detector response to the $\tau$-lepton decay products are based on simulation. This hybrid sample is referred to as embedded data in the following. A detailed description of the embedding procedure can be found in Ref. [40].

The *W*+jets, $Z/\gamma ^{*}\rightarrow \mu \mu$ and $Z/\gamma ^{*}\rightarrow ee$ backgrounds are modelled by the ALPGEN [41] event generator interfaced with PYTHIA8 [42] to provide the parton showering, hadronisation and the modelling of the underlying event. The backgrounds with top quarks are modelled by the POWHEG [43–45] (for $t\bar{t}$, *Wt* and *s*-channel single-top production) and AcerMC [46] (*t*-channel single-top production) event generators interfaced with PYTHIA8. The ALPGEN event generator interfaced with HERWIG [47] is used to model the *WW* process, and HERWIG is used for the *ZZ* and *WZ* processes.

The events with Higgs bosons produced via *ggH* or VBF processes are generated at next-to-leading-order (NLO) accuracy in QCD with the POWHEG [48] event generator interfaced with PYTHIA8 to provide the parton showering, hadronisation and the modelling of the underlying event. The associated production (*ZH* and *WH*) samples are simulated using PYTHIA8. All events with Higgs bosons are produced with a mass of $m_{H}=125$ GeV assuming the narrow width approximation and normalised to cross sections calculated at next-to-next-to-leading order (NNLO) in QCD [49–51]. The SM $H\rightarrow \tau \tau$ decays are simulated by PYTHIA8; the other SM decays of the Higgs boson are negligible. The LFV Higgs boson decays are modelled by the EvtGen [52] event generator according to the phase-space model. In the $H\rightarrow \mu \tau$ and $H\rightarrow e\tau$ decays, the $\tau$-lepton decays are treated as unpolarised because the left- and right-handed $\tau$-lepton polarisation states are produced at equal rates. Finally, the LFV *Z* boson decays are simulated with PYTHIA8 assuming an isotropic decay. The width of the *Z* boson is set to its measured value [20].

For all simulated samples, the decays of $\tau$-leptons are modelled with TAUOLA [53] and the propagation of particles through the ATLAS detector is simulated with GEANT4 [54, 55]. The effect of multiple proton–proton collisions in the same or nearby beam bunch crossings is accounted for by overlaying additional minimum-bias events. Simulated events are weighted so that the distribution of the average number of interactions per bunch crossing matches that observed in data.

Background contributions due to non-prompt leptons in the $\tau _{\mathrm {lep}}$ channel and multi-jet events in the $\tau _{\mathrm {had}}$ channel are estimated using data-driven techniques described in Sects. 4.2 and 5.2.

## Search for $H\rightarrow e\tau$ decays in the $\tau _{\mathrm {had}}$ channel

The search for the LFV $H\rightarrow e\tau$ decays in the $\tau _{\mathrm {had}}$ channel follows exactly the same analysis strategy and utilises the same background estimation techniques as those used in the ATLAS search for the LFV $H\rightarrow \mu \tau$ decays in the $\tau _{\mathrm {had}}$ channel [22]. The only major difference is that a high-$E_{\text {T}}$ electron is required in the final state instead of a muon. A detailed description of the $H\rightarrow e \tau _{\mathrm {had}}$ analysis is provided in the following sections.

### Event selection and categorisation

Signal $H\rightarrow e\tau$ events in the $e \tau _{\mathrm {had}}$ final state are characterised by the presence of exactly one energetic electron and one $\tau _{\mathrm {had}}$ of opposite-sign (OS) charge as well as moderate $E_{\text {T}}^{\text {miss}}$, which tends to be aligned with the $\tau _{\mathrm {had}}$ direction. Same-sign (SS) charge events are used to control the rates of background contributions. Events with identified muons are rejected. Backgrounds for this signature can be broadly classified into two major categories:

- Events with true electron and $\tau _{\mathrm {had}}$ signatures. These are dominated by the irreducible $Z/\gamma ^{*}\rightarrow \tau \tau$ production with some contributions from the $VV\rightarrow e\tau +X$ (where $V=W,Z$), $t\bar{t}$, single-top and SM $H\rightarrow \tau \tau$ production processes. These events exhibit a very strong charge anti-correlation between the electron and the $\tau _{\mathrm {had}}$. Therefore, the expected number of OS events ($N_{\mathrm {OS}}$) is much larger than the number of SS events ($N_{\mathrm {SS}}$).
- Events with a misidentified $\tau _{\mathrm {had}}$ signature. These are dominated by *W*+jets events with some contribution from multi-jet (many of which have genuine electrons from semileptonic decays of heavy-flavour hadrons), diboson (*VV*), $t\bar{t}$ and single-top events with $N_{\mathrm {OS}}>N_{\mathrm {SS}}$. Additional contributions to this category arise from $Z(\rightarrow ee)$+jets events, where a $\tau _{\mathrm {had}}$ signature can be mimicked by either a jet (no charge correlation) or an electron (strong charge anti-correlation).

**Fig. 1:**
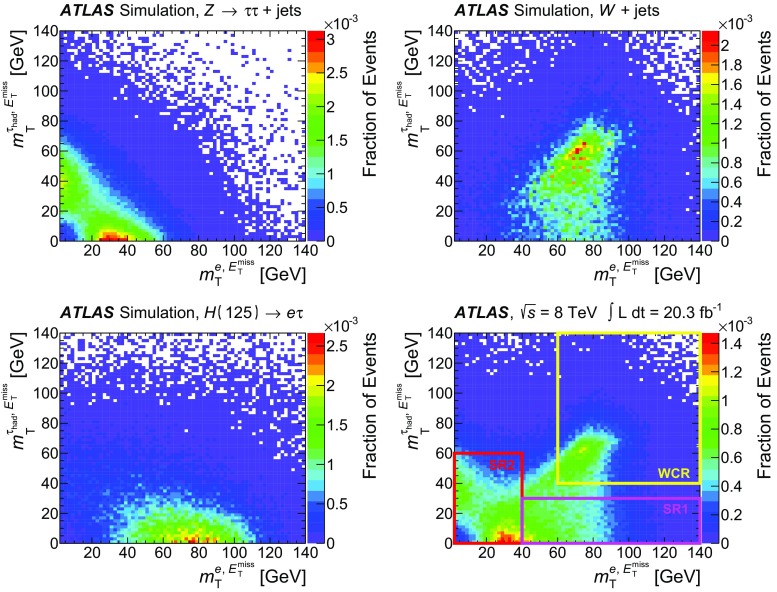
Two-dimensional distributions of the transverse mass of the *e*-$E_{\text {T}}^{\text {miss}}$ system, $m_{\mathrm {T}}^{e,E_{\text {T}}^{\text {miss}}}$, and that of the $\tau _{\mathrm {had}}$-$E_{\text {T}}^{\text {miss}}$ system, $m_{\mathrm {T}}^{ \tau _{\mathrm {had}} ,E_{\text {T}}^{\text {miss}}}$, in simulated $Z/\gamma ^{*}\rightarrow \tau \tau$ (*top left plot*), *W*+jets (*top right plot*), $H\rightarrow e\tau$ signal (*bottom left plot*) and data (*bottom right plot*) events. *Magenta*, *red* and *yellow boxes* on the *bottom right plot* illustrate SR1, SR2, and WCR, respectively. All events used for these distributions are required to have a well-identified electron and $\tau _{\mathrm {had}}$ (as described in text) of opposite charge with $p_{\text {T}} ( \tau _{\mathrm {had}} )>20$ GeV and $E_{\text {T}} (e)>26$ GeV

Events with a misidentified $\tau _{\mathrm {had}}$ tend to have a much softer $p_{\text {T}} ( \tau _{\mathrm {had}} )$ spectrum and a larger angular separation between the $\tau _{\mathrm {had}}$ and $E_{\text {T}}^{\text {miss}}$ directions. These properties are exploited to suppress backgrounds and define signal and control regions. Events with exactly one electron and exactly one $\tau _{\mathrm {had}}$ with $E_{\text {T}} (e)>26$ GeV, $p_{\text {T}} ( \tau _{\mathrm {had}} )>45$ GeV and $|\eta (e)-\eta ( \tau _{\mathrm {had}} )|<2$ form a baseline sample as it represents a common selection for both the signal and control regions. The $|\eta (e)-\eta ( \tau _{\mathrm {had}} )|$ cut has $\sim$99% efficiency for signal and rejects a considerable fraction of multi-jet and *W*+jets events. Similarly as done in Ref. [22], two signal regions are defined using the transverse mass^2^, $m_{\mathrm {T}}$, of the *e*-$E_{\text {T}}^{\text {miss}}$ and $\tau _{\mathrm {had}}$-$E_{\text {T}}^{\text {miss}}$ systems: OS events with $m_{\mathrm {T}}^{e,E_{\text {T}}^{\text {miss}}}>40$ GeV and $m_{\mathrm {T}}^{ \tau _{\mathrm {had}} ,E_{\text {T}}^{\text {miss}}}<30$ GeV form the signal region-1 (SR1), while OS events with $m_{\mathrm {T}}^{e,E_{\text {T}}^{\text {miss}}}<40$ GeV and $m_{\mathrm {T}}^{ \tau _{\mathrm {had}} ,E_{\text {T}}^{\text {miss}}}<60$ GeV form the signal region-2 (SR2). Both regions have similar sensitivity to the signal (see Sect. 4.4). The dominant background in SR1 is *W*+jets, while the $Z/\gamma ^{*}\rightarrow \tau \tau$ and $Z\rightarrow ee$+jets backgrounds dominate in SR2. The modelling of the *W*+jets background is checked in a dedicated control region (WCR) formed by events with $m_{\mathrm {T}}^{e,E_{\text {T}}^{\text {miss}}}>60$ GeV and $m_{\mathrm {T}}^{ \tau _{\mathrm {had}} ,E_{\text {T}}^{\text {miss}}}>40$ GeV. As discussed in detail in Sect. 4.2, the modelling of the $Z/\gamma ^{*}\rightarrow \tau \tau$ and $Z\rightarrow ee$+jets backgrounds is checked in SR2. The choice of $m_{\mathrm {T}}$ cuts to define SR1, SR2 and WCR is motivated by correlations between $m_{\mathrm {T}}^{e,E_{\text {T}}^{\text {miss}}}$ and $m_{\mathrm {T}}^{ \tau _{\mathrm {had}} ,E_{\text {T}}^{\text {miss}}}$ in $H\rightarrow e\tau$ signal and major background (*W*+jets and $Z/\gamma ^{*}\rightarrow \tau \tau$) events, as illustrated in Fig. 1. No events with identified *b*-jets are allowed in SR1, SR2 and WCR. The modelling of the $t\bar{t}$ and single-top backgrounds is checked in a dedicated control region (TCR), formed by events that satisfy the baseline selection and have at least two jets, with at least one being *b*-tagged. Table 2 provides a summary of the event selection criteria used to define the signal and control regions.

The LFV signal is searched for by performing a fit to the mass distribution in data, $m_{e\tau }^{\mathrm {MMC}}$, reconstructed from the observed electron, $\tau _{\mathrm {had}}$ and $E_{\text {T}}^{\text {miss}}$ objects by means of the Missing Mass Calculator [56] (MMC). Conceptually, the MMC is a more sophisticated version of the collinear approximation [57]. The main improvement comes from requiring that the relative orientations of the neutrino and other $\tau$-lepton decay products are consistent with the mass and kinematics of a $\tau$-lepton decay. This is achieved by maximising a probability defined in the kinematically allowed phase-space region. The MMC used in the $H\rightarrow \tau \tau$ analysis [35] is modified to take into account that there is only one neutrino from a hadronic $\tau$-lepton decay in LFV $H\rightarrow e\tau$ events. For a Higgs boson with $m_{H}=125$ GeV, the reconstructed $m_{e\tau }^{\mathrm {MMC}}$ distribution has a roughly Gaussian shape with a full width at half maximum of $\sim$19 GeV. The analysis is performed “blinded” in the 110 GeV$<m_{e\tau }^{\mathrm {MMC}}<$150 GeV regions of SR1 and SR2, which contain 93.5 and 95% of the expected signal events in SR1 and SR2, respectively. The event selection and the analysis strategy are defined without looking at the data in these blinded regions.

**Table 2:** Summary of the event selection criteria used to define the signal and control regions (see text)

Criterion	SR1	SR2	WCR	TCR
$E_{\text {T}} (e)$	>26 GeV	>26 GeV	>26 GeV	>26 GeV
$p_{\text {T}} ( \tau _{\mathrm {had}} )$	>45 GeV	>45 GeV	>45 GeV	>45 GeV
$|\eta (e)-\eta ( \tau _{\mathrm {had}} )|$	<2	<2	<2	<2
$m_{\mathrm {T}}^{e,E_{\text {T}}^{\text {miss}}}$	>40 GeV	<40 GeV	>60 GeV	–
$m_{\mathrm {T}}^{ \tau _{\mathrm {had}} ,E_{\text {T}}^{\text {miss}}}$	<30 GeV	<60 GeV	>40 GeV	–
$N_{\mathrm {jet}}$	–	–	–	$\ge$2
$N_{b{\text {-}}\mathrm {jet}}$	0	0	0	$\ge$1

### Background estimation

The background estimation method takes into account the background properties and composition discussed in Sect. 4.1. It also relies on the observation that the shape of the $m_{e\tau }^{\mathrm {MMC}}$ distribution for the multi-jet background is the same for OS and SS events. This observation was made using a dedicated control region, MJCR, with an enhanced contribution from the multi-jet background. Events in this control region are required to meet all criteria for SR1 and SR2 with the exception of the requirement on $|\eta (e)-\eta ( \tau _{\mathrm {had}} )|$, which is reversed: $|\eta (e)-\eta ( \tau _{\mathrm {had}} )|>2$. Therefore, the total number of OS background events, $N_{\mathrm {OS}}^{\mathrm {bkg}}$ in each bin of the $m_{e\tau }^{\mathrm {MMC}}$ (or any other) distribution in SR1 and SR2 can be obtained according to the following formula:

1 $$\begin{aligned} N_{\mathrm {OS}}^{\mathrm {bkg}} =r_{\mathrm {QCD}}\cdot N_{\mathrm {SS}}^{\mathrm {data}} + \sum _{\mathrm {bkg}{\text {-}}i} N_{\mathrm {OS-SS}}^{\mathrm {bkg}{\text {-}}i}, \end{aligned}$$

where the individual terms are described below. $N_{\mathrm {SS}}^{\mathrm {data}}$ is the number of SS data events, which contains significant contributions from *W*+jets events, multi-jet and other backgrounds. The fractions of multi-jet background in SS data events inside the 110 GeV$< m_{e\tau }^{\mathrm {MMC}}<$150 GeV mass window are $\sim$27 and $\sim$64% in SR1 and SR2, respectively. The contributions $N_{\mathrm {OS-SS}}^{\mathrm {bkg}{\text {-}}i}=N_{\mathrm {OS}}^{\mathrm {bkg}{\text {-}}i}-r_{\mathrm {QCD}}\cdot N_{\mathrm {SS}}^{\mathrm {bkg}{\text {-}}i}$ are *add-on* terms for the different background components (where bkg-*i* indicates the $i^{\mathrm {th}}$ background source: $Z\rightarrow \tau \tau$, $Z\rightarrow ee$, *W*+jets, *VV*, $H\rightarrow \tau \tau$ and events with *t*-quarks), which also account for components of these backgrounds already included in SS data events.^3^ The factor $r_{\mathrm {QCD}}=N_{\mathrm {OS}}^{\mathrm {multi{\text {-}}jet}}/N_{\mathrm {SS}}^{\mathrm {multi{\text {-}}jet}}$ accounts for potential differences in flavour composition (and, as a consequence, in jet $\rightarrow \tau _{\mathrm {had}}$ misidentification rates) of final-state jets introduced by the same-sign or opposite-sign charge requirements. The value of $r_{\mathrm {QCD}}= 1.0\pm 0.13$ is obtained from a multi-jet enriched control region in data using a method discussed in Ref. [58]. This sample is obtained by selecting events with $E_{\text {T}}^{\text {miss}} <15$ GeV, $m_{\mathrm {T}}^{e,E_{\text {T}}^{\text {miss}}}<30$ GeV, removing the isolation criteria of the electron candidate and using the *loose* identification criteria for the $\tau _{\mathrm {had}}$ candidate [34]. The systematic uncertainty on $r_{\mathrm {QCD}}$ is estimated by varying the selection cuts described above. The obtained value of $r_{\mathrm {QCD}}$ is also verified in the MJCR region, which has a smaller number of events but where the electron and $\tau _{\mathrm {had}}$ candidates pass the same identification requirements as events in SR1 and SR2.

The data and simulation samples used for the modelling of background processes are described in Sect. 3. A discussion of each background source is provided below.

The largely irreducible $Z/\gamma ^{*}\rightarrow \tau \tau$ background is modelled by the embedded data sample described in Sect. 3. The $Z/\gamma ^{*}\rightarrow \tau \tau$ normalisation is a free parameter in the final fit to data and it is mainly constrained by events with 60 GeV$<$ $m_{e\tau }^{\mathrm {MMC}}$ $<$90 GeV in SR2.

Events due to the *W*+jets background are mostly selected when the $\tau _{\mathrm {had}}$ signature is mimicked by jets. This background is estimated from simulation, and the WCR region is used to check the modelling of the *W*+jets kinematics and to obtain separate normalisations for OS and SS *W*+jets events. The difference in these two normalisations happens to be statistically significant. An additional overall normalisation factor for the $N_{\mathrm {OS-SS}}^{W+\mathrm {jets}}$ term in Eq. (1) is introduced as a free parameter in the final fit in SR1. By studying WCR events and SR1 events with $m_{e\tau }^{\mathrm {MMC}}>150$ GeV (dominated by *W*+jets background), it is also found that an $m_{e\tau }^{\mathrm {MMC}}$ shape correction, which depends on the number of jets, $p_{\text {T}} ( \tau _{\mathrm {had}} )$ and $|\eta (e)-\eta ( \tau _{\mathrm {had}} )|$, needs to be applied in SR1. This correction is derived from SR1 events with $m_{e\tau }^{\mathrm {MMC}}>150$ GeV and it is applied to events with any value of $m_{e\tau }^{\mathrm {MMC}}$. The corresponding modelling uncertainty is set to be 50% of the difference of the $m_{e\tau }^{\mathrm {MMC}}$ shapes obtained after applying the SR1-based and WCR-based shape corrections. The size of this uncertainty depends on $m_{e\tau }^{\mathrm {MMC}}$ and it is as large as ±10% for *W*+jets events with $m_{e\tau }^{\mathrm {MMC}}< 150$ GeV. In the case of SR2, good modelling of the $N_{\mathrm {jet}}$, $p_{\text {T}} ( \tau _{\mathrm {had}} )$ and $|\eta (e)-\eta ( \tau _{\mathrm {had}} )|$ distributions suggests that such a correction is not needed. However, a modelling uncertainty in the $m_{e\tau }^{\mathrm {MMC}}$ shape of the *W*+jets background in SR2 is set to be 50% of the difference between the $m_{e\tau }^{\mathrm {MMC}}$ shape obtained without any correction and the one obtained after applying the correction derived for SR1 events. The size of this uncertainty is below 10% in the 110 GeV$< m_{e\tau }^{\mathrm {MMC}}<$150 GeV region, which contains most of the signal events. It was also checked that applying the same correction in SR2 as in SR1 would affect the final result by less than 4% (see Sect. 6). The modelling of jet fragmentation and the underlying event has a significant effect on the estimate of the jet $\rightarrow \tau _{\mathrm {had}}$ misidentification rate in different regions of the phase space and has to be accounted for with a corresponding systematic uncertainty. To estimate this effect, the analysis was repeated using a sample of *W*+jets events modelled by ALPGEN interfaced with the HERWIG event generator. Differences in the *W*+jets predictions in SR1 and SR2 are found to be ±12 and ±15%, respectively, and are taken as corresponding systematic uncertainties.

In the case of the $Z\rightarrow ee$ background, there are two components: events in which an electron mimics a $\tau _{\mathrm {had}}$ ($e\rightarrow \tau _{\mathrm {had}} ^{\mathrm {misid}} {}$) and events in which a jet mimics a $\tau _{\mathrm {had}}$ (jet$\rightarrow \tau _{\mathrm {had}} ^{\mathrm {misid}} {}$). In the first case, the shape of the $Z\rightarrow ee$ background is obtained from simulation. Corrections from data, derived from dedicated tag-and-probe studies [59], are also applied to account for the variation in the $e\rightarrow \tau _{\mathrm {had}} ^{\mathrm {misid}} {}$ misidentification rate as a function of $\eta$. The normalisation of this background component is a free parameter in the final fit to data and it is mainly constrained by events with 90 GeV$<$ $m_{e\tau }^{\mathrm {MMC}}$ $<$110 GeV in SR2. For the $Z\rightarrow ee$ background where a jet is misidentified as a $\tau _{\mathrm {had}}$ candidate and one of the electrons does not pass the electron identification criteria described in Sect. 2, the normalisation factor and shape corrections, which depend on the number of jets, $p_{\text {T}} ( \tau _{\mathrm {had}} )$ and $|\eta (e)-\eta ( \tau _{\mathrm {had}} )|$, are derived using events with two identified OS electrons with an invariant mass, $m_{ee}$, in the range of 80–100 GeV. Since this background does not have an OS–SS charge asymmetry, a single correction factor is derived for OS and SS events. Half the difference between the $m_{e\tau }^{\mathrm {MMC}}$ shape with and without this correction is taken as the corresponding systematic uncertainty.

The TCR is used to check the modelling and to obtain normalisations for OS and SS events with top quarks. The normalisation factors obtained in the TCR are extrapolated into SR1 and SR2, where $t\bar{t}$ and single-top events may have different properties. To estimate the uncertainty associated with such an extrapolation, the analysis is repeated using the MC@NLO [60] event generator instead of POWHEG for $t\bar{t}$ production.^4^ This uncertainty is found to be ±8% (±14%) for backgrounds with top quarks in SR1 (SR2).

The background due to diboson (*WW*, *ZZ* and *WZ*) production is estimated from simulation, normalised to the cross sections calculated at NLO in QCD [61]. Finally, the SM $H\rightarrow \tau \tau$ events also represent a small background in this search. This background is estimated from simulation and normalised to the cross sections calculated at NNLO in QCD [49–51]. All other SM Higgs boson decays constitute negligible backgrounds for the LFV signature.

**Fig. 2:**
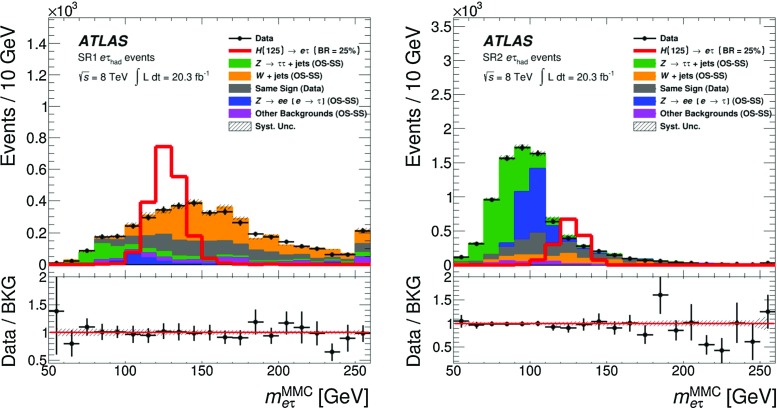
Distributions of the mass reconstructed by the Missing Mass Calculator, $m_{e\tau }^{\mathrm {MMC}}$, in SR1 (*left*) and SR2 (*right*). The background distributions are determined in a global fit (described in Sect. 4.4). The signal distribution corresponds to Br($H\rightarrow e\tau$) = 25%. The *bottom panel* of each sub-figure shows the ratio of the observed data to the estimated background. Very small backgrounds due to single top, $t\bar{t}$, *VV*, $Z\rightarrow ee(\text {jet}\rightarrow \tau _{\mathrm {had}} ^{\mathrm {misid}} {})$ and $H\rightarrow \tau \tau$ events are combined in a single background component labelled as “Other Backgrounds”. The *grey band* for the ratio illustrates post-fit systematic uncertainties in the background prediction. The statistical uncertainties in the background predictions and data are added in quadrature for the ratios. The last bin in each distribution contains events with $m_{e\tau }^{\mathrm {MMC}}>$ 250 GeV

Figure 2 shows the $m_{e\tau }^{\mathrm {MMC}}$ distributions for data and the predicted backgrounds in each of the signal regions. The backgrounds are estimated using the method described above and their normalisations are obtained in a global fit described in Sect. 4.4. The signal acceptance times efficiencies for passing the SR1 or SR2 selection requirements are 1.8 and 1.4%, respectively, and the combined efficiency is 3.2%. The numbers of observed events in the data as well as the signal and background predictions in the mass region 110 GeV$< m_{e\tau }^{\mathrm {MMC}}<$150 GeV can be found in Table 3.

**Table 3:** Data yields, signal and post-fit OS–SS background predictions (see Eq. (1)) for the 110 GeV$< m_{e\tau }^{\mathrm {MMC}}<$150 GeV region. The signal predictions are given for Br($H\rightarrow e\tau$) = 1.0%. The background predictions are obtained from the combined fit to SR1, SR2, WCR and TCR. The post-fit values of systematic uncertainties are provided for the background predictions. For the total background, all correlations between various sources of systematic uncertainties and backgrounds are taken into account. The quoted uncertainties represent the statistical (first) and systematic (second) uncertainties, respectively

	SR1			SR2		
LFV signal (Br($H\rightarrow e\tau$) = 1.0%)	75	±1	±8	59	±1	±8
*W*+jets	740	±80	±110	370	±60	±70
Same-Sign events	390	±20	±60	570	±30	±80
$Z\rightarrow \tau \tau$	116	±8	±11	245	±11	±20
*VV* and $Z\rightarrow ee(jet\rightarrow \tau _{\mathrm {had}} ^{\mathrm {misid}} {})$	71	±31	±30	60	±20	±40
$Z\rightarrow ee(e\rightarrow \tau _{\mathrm {had}} ^{\mathrm {misid}} {})$	69	±17	±11	320	±40	±40
$t\bar{t}$ and single top	18	±5	±4	10.2	±2.6	±2.2
$H\rightarrow \tau \tau$	4.6	±0.2	±0.7	10.5	±0.3	±1.5
Total background	1410	±90	±70	1590	±80	± 70
Data	1397		1501			

### Systematic uncertainties

The numbers of signal and background events and the shapes of corresponding $m_{e\tau }^{\mathrm {MMC}}$ distributions are affected by systematic uncertainties. They are discussed below and changes in event yields are provided for major sources of uncertainties. For all uncertainties, the effects on both the total signal and background predictions and on the shape of the $m_{e\tau }^{\mathrm {MMC}}$ distribution are evaluated. Unless otherwise mentioned, all sources of experimental uncertainties are treated as fully correlated across signal and control regions in the final fit which is discussed in Sect. 4.4.

The largest systematic uncertainties arise from the normalisation (±12% uncertainty) and modelling of the *W*+jets background. The uncertainties on the *W*+jets normalisation and $m_{e\tau }^{\mathrm {MMC}}$ shape corrections are treated as uncorrelated between SR1 and SR2. The uncertainties in $r_{\mathrm {QCD}}$ (±13%) and in the normalisation (±13%) and modelling of $Z\rightarrow \tau \tau$ also play an important role. The normalisation uncertainty (±7%) for the $Z\rightarrow ee$ (with $e\rightarrow \tau _{\mathrm {had}} ^{\mathrm {misid}} {}$) background has a limited impact on the sensitivity because of a good separation of the signal and $Z\rightarrow ee$ peaks in the $m_{e\tau }^{\mathrm {MMC}}$ distribution. The other major sources of experimental uncertainty, affecting both the shape and normalisation of signal and backgrounds, are the uncertainty in the $\tau _{\mathrm {had}}$ energy scale [34], which is measured with ±(2–4)% precision (depending on $p_{\text {T}}$ and decay mode of the $\tau _{\mathrm {had}}$ candidate), and uncertainties in the embedding method used to model the $Z\rightarrow \tau \tau$ background [35]. Less significant sources of experimental uncertainty, affecting the shape and normalisation of signal and backgrounds, are the uncertainty in the jet energy scale [37, 62] and resolution [63]. The uncertainties in the $\tau _{\mathrm {had}}$ energy resolution, the energy scale and resolution of electrons, and the scale uncertainty in $E_\mathrm {T}^\mathrm {miss}$ due to the energy in calorimeter cells not associated with physics objects are taken into account; however, they are found to be only ±(1–2$\%$). The following experimental uncertainties primarily affect the normalisation of signal and backgrounds: the ±2.8% uncertainty in the integrated luminosity [64], the uncertainty in the $\tau _{\mathrm {had}}$ identification efficiency [34], which is measured to be ±(2–3)% for 1-prong and ±(3–5)% for 3-prong decays(where the range reflects the dependence on $p_{\text {T}}$ of the $\tau _{\mathrm {had}}$ candidate), the ±2.1% uncertainty for triggering, reconstructing and identifying electrons [33], and the ±2% uncertainty in the *b*-jet tagging efficiency [38].

Theoretical uncertainties are estimated for the Higgs boson production and for the *VV* background, which are modelled with the simulation and are not normalised to data in dedicated control regions. Uncertainties due to missing higher-order QCD corrections in the production cross sections are found to be [65] ±10.1$\%$ (±7.8$\%$) for the Higgs boson production via *ggH* in SR1 (SR2), ±1$\%$ for the $Z\rightarrow ee$ background and for VBF and *VH* Higgs boson production, and ±5% for the *VV* background. The systematic uncertainties due to the choice of parton distribution functions used in the simulation are evaluated based on the prescription described in Ref. [65] and the following values are used in this analysis: ±7.5$\%$ for the Higgs boson production via *ggH*, ±2.8$\%$ for the VBF and *VH* Higgs boson production, and ±4$\%$ for the *VV* background. Finally, an additional ±5.7% systematic uncertainty [65] on Br($H\rightarrow \tau \tau$) is applied to the SM $H\rightarrow \tau \tau$ background.

### Results of the search for LFV $H\rightarrow e\tau$ decays in the $\tau _{\mathrm {had}}$ channel

**Fig. 3:**
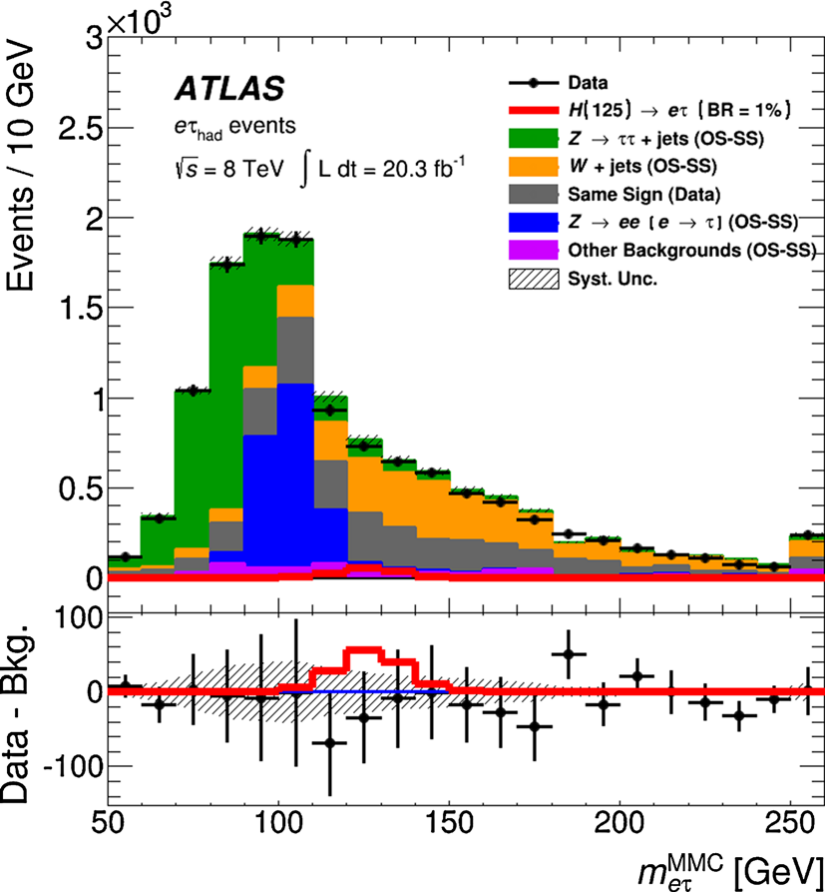
Post-fit combined $m_{e\tau }^{\mathrm {MMC}}$ distribution obtained by adding individual distributions in SR1 and SR2. In the lower part of the figure, the data are shown after subtraction of the estimated backgrounds. The *grey band* in the *bottom panel* illustrates the post-fit systematic uncertainties in the background prediction. The statistical uncertainties for data and background predictions are added in quadrature in the bottom part of the figure. The signal is shown assuming Br$(H\rightarrow e\tau )=1.0\%$. Very small backgrounds due to single top, $t\bar{t}$, *VV*, $Z\rightarrow ee(jet\rightarrow \tau _{\mathrm {had}} ^{\mathrm {misid}} {})$ and $H\rightarrow \tau \tau$ events are combined in a single background component labelled as “Other Backgrounds”. The last bin of the distribution contains events with $m_{e\tau }^{\mathrm {MMC}}>$250 GeV

A simultaneous binned maximum-likelihood fit is performed on the $m_{e\tau }^{\mathrm {MMC}}$ distributions in SR1 and SR2 and on event yields in WCR and TCR to extract the LFV branching ratio Br($H\rightarrow e\tau$). The fit exploits the control regions and the distinct shapes of the *W*+jets, $Z\rightarrow \tau \tau$ and $Z\rightarrow ee$ backgrounds in the signal regions to constrain some of the systematic uncertainties. This increases the sensitivity of the analysis. The post-fit $m_{e\tau }^{\mathrm {MMC}}$ distributions in SR1 and SR2 are shown in Fig. 2, and the combined $m_{e\tau }^{\mathrm {MMC}}$ distribution for both signal regions is presented in Fig. 3. Figure 2 illustrates the level of agreement between data and background expectations in SR1 and SR2. No statistically significant deviations of the data from the predicted background are observed. An upper limit on the LFV branching ratio Br($H\rightarrow e\tau$) for a Higgs boson with $m_{H}=125$ GeV is set using the CL$_s$ modified frequentist formalism [66] with the test statistic based on the profile likelihood ratio [67]. The observed and the median expected 95% CL upper limits are $1.81$% and $2.07^{+0.82}_{-0.58}$%, respectively. Table 6 provides a summary of all results, including the results of the ATLAS search for the LFV $H\rightarrow \mu \tau$ decays [22].

## Search for $H\rightarrow e\tau /\mu \tau$ decays in the $\tau _{\mathrm {lep}} {}$ channel

In the $\tau _{\mathrm {lep}}$ channel the background estimate is based on the data-driven method developed in Ref. [29]. This method is sensitive only to the difference between Br($H\rightarrow \mu \tau$) and Br($H\rightarrow e\tau$), and it is based on the premise that the kinematic properties of the SM background are to a good approximation symmetric under the exchange $e\leftrightarrow {}\mu$.

### Event selection and signal region definition

Events selected in the $\tau _{\mathrm {lep}}$ channel must contain exactly two opposite-sign leptons, one an electron and the other a muon. The lepton with the higher $p_{\text {T}}$ is indicated by $\ell _1$ and the other by $\ell _2$. Additional kinematic criteria, based on the $p_{\text {T}}$ difference between the two leptons and on the angular separations between the leptons and the missing transverse momentum, are applied to suppress the SM background events, which are mainly due to the production of $Z/\gamma ^{*}\rightarrow \tau \tau$ and of diboson (*VV*) events. Two mutually exclusive signal regions are defined: one with no central ($|\eta |<2.4$) light-flavour jets, $\mathrm {SR_{noJets}}$, and the other with one or more central light-flavoured jets, $\mathrm {SR_{withJets}}$. The kinematic criteria defining each signal region, summarised in Table 4, are optimised following two guidelines. The first one is to maximise the signal-to-background ratio. The second one is to have, in each signal region, enough events to perform the data-driven background estimation described in Sect. 5.2.

**Table 4:** Summary of the selection criteria used to define the signal regions in the $\tau _{\mathrm {lep}}$ channel (see text)

	$\mathrm {SR_{noJets}}$	$\mathrm {SR_{withJets}}$
Light leptons	$e^{\pm }\mu ^{\mp }$	$e^{\pm }\mu ^{\mp }$
$\tau _{\mathrm {had}}$ leptons	veto	veto
Central jets	0	$\ge 1$
*b*-jets	0	0
$p_{\text {T}} ^{\ell _1}$	$\ge 35\,\mathrm{GeV}$	$\ge 35\,\mathrm{GeV}$
$p_{\text {T}} ^{\ell _2}$	$\ge 12\,\mathrm{GeV}$	$\ge 12\,\mathrm{GeV}$
$\left| \eta ^{e}\right|$	$\le 2.4$	$\le 2.4$
$\left| \eta ^{\mu }\right|$	$\le 2.4$	$\le 2.4$
$\Delta \phi (\ell _2,E_{\text {T}}^{\text {miss}})$	$\le 0.7$	$\le 0.5$
$\Delta \phi (\ell _1,\ell _2)$	$\ge 2.3$	$\ge 1.0$
$\Delta \phi (\ell _1,E_{\text {T}}^{\text {miss}})$	$\ge 2.5$	$\ge 1.0$
$\Delta p_{\text {T}} (\ell _1,\ell _2)$	$\ge 7\,\mathrm{GeV}$	$\ge 1\,\mathrm{GeV}$

The final discriminant used in the $\tau _{\mathrm {lep}}$ channel is the collinear mass $\mathrm {m_{coll}}$ defined as:

2 $$\begin{aligned} m_\mathrm{coll}{} = \sqrt{2p_{\mathrm {T}}^{\ell {}_{1}}{} (p_{\mathrm {T}}^{\ell {}_{2}}{}+E_{\text {T}}^{\text {miss}} {}) (\cosh {}\Delta \eta -\cos \Delta \phi )}. \end{aligned}$$

This quantity is the invariant mass of two massless particles, $\tau {}$ and $\ell _1$, computed with the approximation that the decay products of the $\tau {}$ lepton, $\ell _2$ and neutrinos, are collinear to the $\tau {}$, and that the $E_{\text {T}}^{\text {miss}}$ originates from the $\nu {}$. In the $H \rightarrow \mu {}\tau {}$ ($H \rightarrow {}e\tau {}$) decay, $\ell _1$ is the muon (electron) and $\ell _2$ is the electron (muon). The differences in rapidity and azimuthal angle between $\ell _1$ and $\ell _2$ are indicated by $\Delta \eta {}$ and $\Delta \phi {}$. More sophisticated kinematic variables, such as MMC, do not significantly improve the sensitivity of the $\tau _{\mathrm {lep}}$ channel.

### Background estimation

For simplicity, the symmetry method is illustrated here assuming a $H \rightarrow \mu {}\tau {}$ signal. The same procedure, but with *e* and $\mu {}$ exchanged, is valid under the $H \rightarrow {}e\tau {}$ assumption. The symmetry method is based on the following two premises:

1. SM processes result in data that are symmetric under the exchange of prompt electrons with prompt muons to a good approximation. In other words, the kinematic distributions of prompt electrons and prompt muons are approximately the same;^5^
2. flavour-violating decays of the Higgs boson break this symmetry.

Dilepton events in the dataset are divided into two mutually exclusive samples:

- $\varvec{\mu {}e}$ **sample**: $\ell _1$ is the muon and $\ell _2$ is the electron ($p_{\text {T}} {}^\mu \ge p_{\text {T}} {}^e$)
- $\varvec{e\mu {}}$ **sample**: $\ell _1$ is the electron and $\ell _2$ is the muon ($p_{\text {T}} {}^e > p_{\text {T}} {}^\mu$)

With these assumptions, the SM background is split equally between the two samples. The $H \rightarrow \mu {}\tau {}$ signal, however, is present only in the $\mu e$ sample because the $p_{\text {T}}$ spectrum of electrons from $H \rightarrow \mu {}\tau {}$ decays is softer then the muon $p_{\text {T}}$ spectrum. The number of $H \rightarrow \mu {}\tau {}$ events in the $e\mu$ sample is negligible with the selection criteria described in Sect. 5.1.

For SM events the distributions of kinematic variables in the two samples are the same with good approximation. In particular, the collinear mass distribution differs between the two samples only for the narrow signal peak. The peak, present only in the distribution of the $\mu e$ sample, is on top of the SM background, which, to a good approximation, can be modelled from the $e\mu$ collinear mass distribution.

#### Asymmetries in the SM background

Although the $e\mu$-$\mu e$ symmetry hypothesis is a good starting assumption, there are effects that can invalidate it and that need to be accounted for. The first effect is due to events containing misidentified and non-prompt leptons, together referred to as *non-prompt* in the following. These leptons originate from misidentified jets or from hadronic decays within jets. They contribute differently to the $\mu e$ and $e\mu$ samples because the origin of the non-prompt lepton is different for electrons and for muons. The second effect originates from the different dependencies on $p_{\text {T}}$ and $|\eta |$ that the trigger efficiency and reconstruction efficiency can have for electrons and muons. The non-prompt effect is accounted for by estimating the non-prompt background separately from the other backgrounds. The efficiency effect is accounted for by scaling the $m_\mathrm{coll}$ distribution of the $e\mu$ sample with a scale factor parameterised as a function of the sub-leading lepton $p_{\text {T}}$ , $p_{\mathrm {T}}^{\ell {}_{2}}$. As shown in Sect. 5.5, the $e\mu$-$\mu e$ symmetry is restored when these two effects are taken into account. Smaller effects, which might depend on other parameters such as $\eta$ or $p_{\mathrm {T}}^{\ell {}_{1}}$, are found to be negligible.

*Events containing non-prompt leptons* The background contribution due to non-prompt leptons is estimated with the matrix method described in Refs. [68, 69], which relies on the difference in identification efficiency between prompt and non-prompt leptons. Two lepton categories are defined: tight leptons, which must satisfy all the lepton identification criteria described in Sect. 2, and loose leptons, which are not required to satisfy the primary vertex and isolation criteria. By measuring separately for prompt and non-prompt leptons the tight-to-loose lepton efficiencies, defined as the fraction of loose leptons that are also tight, one can determine the non-prompt background contribution from the number of data events that have two leptons that are either loose or tight. The efficiencies for prompt and non-prompt leptons, parameterised as a function of $p_{\text {T}}$ and $\eta$, are derived from data with the tag-and-probe method. Prompt efficiencies are derived from an opposite-sign sample enriched in $Z\rightarrow {}e^{\pm }e^{\mp }$ and $Z\rightarrow {}\mu ^{\pm }\mu ^{\mp }$. Non-prompt efficiencies are derived from a same-sign sample ($\mu ^{\pm }e^{\pm }$ or $\mu ^{\pm }\mu ^{\pm }$) where the muon is the tag lepton.

*Asymmetry induced by the different trigger and reconstruction efficiency of electrons and muons* The efficiency to trigger on and reconstruct an $e\mu$ event, $\varepsilon ^{e\mu }$, is different from the one of a $\mu e$ event, $\varepsilon ^{\mu {}e}$. These two efficiencies can be expressed as a function of the $p_{\text {T}}$ of the two leptons:

$$\begin{aligned} \varepsilon ^{\mu e}= \varepsilon ^{\mu e}_{\text {trig.}}\left( p_{\text {T}} ^{\ell _{2}=e}\right) \times \varepsilon ^{\mu }_{\text {reco.}}\left( p_{\text {T}} ^{\ell _1=\mu }\right) \times \varepsilon ^{e}_{\text {reco.}}\left( p_{\text {T}} ^{\ell _2=e}\right) \end{aligned}$$

$$\begin{aligned} \varepsilon ^{e \mu }= \varepsilon ^{e \mu }_{\text {trig.}}\left( p_{\text {T}} ^{\ell _{2}=\mu }\right) \times \varepsilon ^{e}_{\text {reco.}}\left( p_{\text {T}} ^{\ell _1=e}\right) \times \varepsilon ^{\mu }_{\text {reco.}}\left( p_{\text {T}} ^{\ell _2=\mu }\right) . \end{aligned}$$

In this search, the leading lepton is required to have $p_{\mathrm {T}}^{\ell {}_{1}}{}>35~\mathrm{GeV}{}$, which is on the plateau region of the trigger and reconstruction efficiencies. Hence the ratio of the efficiencies can be approximated as:

$$\begin{aligned} \frac{\varepsilon ^{\mu e}}{\varepsilon ^{e \mu }}&= \frac{ \varepsilon ^{\mu e}_{\text {trig.}}\left( p_{\text {T}} ^{\ell _{2}}\right) \varepsilon ^{\mu }_{\text {reco.}}\left( p_{\text {T}} ^{\ell _1}\right) \varepsilon ^{e}_{\text {reco.}}\left( p_{\text {T}} ^{\ell _2}\right) }{ \varepsilon ^{e\mu }_{\text {trig.}}\left( p_{\text {T}} ^{\ell _{2}}\right) \varepsilon ^{e}_{\text {reco.}}\left( p_{\text {T}} ^{\ell _1}\right) \varepsilon ^{\mu }_{\text {reco.}}\left( p_{\text {T}} ^{\ell _2}\right) } \\&= \frac{ \varepsilon ^{\mu e}_{\text {trig.}}\left( p_{\text {T}} ^{\ell _{2}}\right) \varepsilon ^{e}_{\text {reco.}}\left( p_{\text {T}} ^{\ell _2}\right) }{ \varepsilon ^{e\mu }_{\text {trig.}}\left( p_{\text {T}} ^{\ell _{2}}\right) \varepsilon ^{\mu }_{\text {reco.}}\left( p_{\text {T}} ^{\ell _2}\right) }\times \frac{ \varepsilon ^{\mu }_{\text {reco.}}\left( p_{\text {T}} ^{\ell _1}\right) }{ \varepsilon ^{e}_{\text {reco.}}\left( p_{\text {T}} ^{\ell _1}\right) } \\&= f\left( p_{\mathrm {T}}^{\ell {}_{2}}\right) {}\times \text {Const.} \end{aligned}$$

Therefore, the ratio of the $e\mu$ and $\mu e$ event reconstruction efficiencies can be parameterised as a function of the sub-leading lepton $p_{\text {T}}$ , $f\left( p_{\mathrm {T}}^{\ell {}_{2}}\right)$. Using the fit described in Sect. 5.4, the parameter $f\left( p_{\mathrm {T}}^{\ell {}_{2}}\right)$ is determined in three $p_{\mathrm {T}}^{\ell {}_{2}}$ bins, 12–20, 20–30, and $> 30~\mathrm{GeV}{}$.

### Systematic uncertainties

Using the $e\mu {}$ asymmetry technique, the only systematic uncertainty associated with the background prediction is due to the non-prompt background modelling. This uncertainty has two components: the first one is the limited number of tag-and-probe events used to extract the prompt and non-prompt efficiencies; the second one is the difference in kinematics, and therefore in sources of non-prompt leptons, between the events used to extract the non-prompt efficiency and the events in the signal regions. This second component is evaluated by measuring the non-prompt efficiencies in subsets of the nominal tag-and-probe sample. The subsets are obtained by applying, one at a time, the kinematic requirements of the signal regions. The ensuing uncertainties in the estimated number of non-prompt events can be as large as 10–50$\%$ for the non-prompt efficiency and $3\%$ for the prompt efficiency, depending on the signal region.

Uncertainties related to the signal prediction are the same ones described in Sect. 4.3 with one minor difference in the uncertainty in the signal cross section due to higher-order QCD corrections. This uncertainty is split into two anticorrelated components: $\pm {}12\%$ in $\mathrm {SR_{withJets}}$ and $\pm {}20\%$ in $\mathrm {SR_{noJets}}$.

### The statistical model

Assuming that the SM background is completely symmetric when exchanging $e\leftrightarrow \mu {}$ , the likelihood function for the collinear mass distribution of the $e\mu$ and $\mu e$ samples can be written as:

3 $$\begin{aligned} L(b_i,\mu ) = \prod \limits _i^{N_{m_\mathrm{coll}{}}} \mathrm{Pois}(n_i\mid b_i) \times \mathrm{Pois}(m_i\mid b_i+\mu s_i), \end{aligned}$$

where $n_i$ $(m_i)$ is the number of $e\mu$ ($\mu e$) events in the *i*-th of the $N_{m_\mathrm{coll}}$ $m_\mathrm{coll}$ bins. The number of background events in the *i*-th $m_\mathrm{coll}$ bin is indicated by $b_i$, and $s_i$ is the number of $H \rightarrow \mu {}\tau {}$ events in the *i*-th mass bin. The number of signal events $\sum \limits _{i}s_i$ is normalised to a branching ratio $\text{ Br }(H \rightarrow \mu {}\tau {}{})=1\%$, multiplied by a signal strength $\mu$. The likelihood for the $m_\mathrm{coll}$ distributions with a $H \rightarrow {}e\tau {}$ signal can be defined in a similar way. The contributions due to non-prompt leptons add to the $e\mu$ and $\mu e$ terms and they are denoted by $N_i^{\text {np}}$ and $M_i^{\text {np}}$, along with their uncertainties, $\sigma _{N_i^{\text {np}}}$ and $\sigma _{M_i^{\text {np}}}$. The numbers of non-prompt events in each bin, $N_i^{\text {np}}$ and $M_i^{\text {np}}$, are treated as Gaussian nuisance parameters.

The $f\left( p_{\mathrm {T}}^{\ell {}_{2}}\right)$ correction, described in Sect. 5.2, is implemented by performing the fit separately in $N_{p_{\mathrm {T}}^{\ell {}_{2}}{}}=3$ $p_{\mathrm {T}}^{\ell {}_{2}}$ bins, labelled with the index *j*. The corrective scale factor $A_j$, corresponding to the $f\left( p_{\mathrm {T}}^{\ell {}_{2}}\right)$ value in the $m_\mathrm{coll}$ bin *i* and $p_{\mathrm {T}}^{\ell {}_{2}}$ bin *j*, multiplies the $e\mu$ yield $b_{ij}$. These scale factors are treated in the statistical model as unconstrained nuisance parameters.

Adding up the symmetric contribution ($b_{ij}$), the non-prompt contributions ($N_{ij}^{\text {np}}$ and $M_{ij}^{\text {np}}$), the $f\left( p_{\mathrm {T}}^{\ell {}_{2}}\right) {}$ correction, and the signal contribution ($s_{ij}$), the likelihood is written as:

4 $$\begin{aligned} L(\mu , b_{ij}, n_{ij}^{\text {np}}, m_{ij}^{\text {np}})= & {} \prod \limits _i^{N_{m_\mathrm{coll}{}}} \prod \limits _j^{N_{p_{\mathrm {T}}^{\ell {}_{2}}{}}} \mathrm{Pois}(n_{ij}\mid A_jb_{ij} + n_{ij}^{\text {np}})\nonumber \\&\times \mathrm{Pois}(m_{ij}\mid b_{ij} + m_{ij}^{\text {np}} + \mu s_{ij})\nonumber \\&\times \mathrm{Gaus}(n_{ij}^{\text {np}} | N_{ij}^{\text {np}} , \sigma _{N_{ij}^{\text {np}}})\nonumber \\&\times \mathrm{Gaus}(m_{ij}^{\text {np}} | M_{ij}^{\text {np}} , \sigma _{M_{ij}^{\text {np}}}). \end{aligned}$$

### Background model validation

The symmetry-based method is validated with simulation and with data. The validation with simulated samples is performed by comparing the signal strength measured in the SR with background samples, and with signal samples corresponding to several non-zero LFV branching ratios. The validation with data is performed in a validation region (VR) defined as $\mathrm {SR_{noJets}}$, but with at least one angular requirement reversed, $\Delta \phi (\ell _1,\ell _2)$ or $\Delta \phi (\ell _1,E_{\text {T}}^{\text {miss}})$.

The validation procedure consists of comparing the data, or the sum of the simulated background samples, to the total background estimated from the statistical model. The comparison is done for the $e\mu$ sample and the $\mu e$ one. With the simulated samples, it is also verified that the symmetric background and the $f\left( p_{\mathrm {T}}^{\ell {}_{2}}\right)$ do not depend on the presence of an LFV signal.

Generated pseudo-experiments are used to confirm that the statistical model is unbiased. No significant discrepancy was found between the injected signal strength and its fitted value up to LFV branching ratios of $10\%$.

### Results of the search for LFV $H\rightarrow e\tau /\mu \tau$ decays in the $\tau _{\mathrm {lep}} {}$ channel

**Fig. 4:**
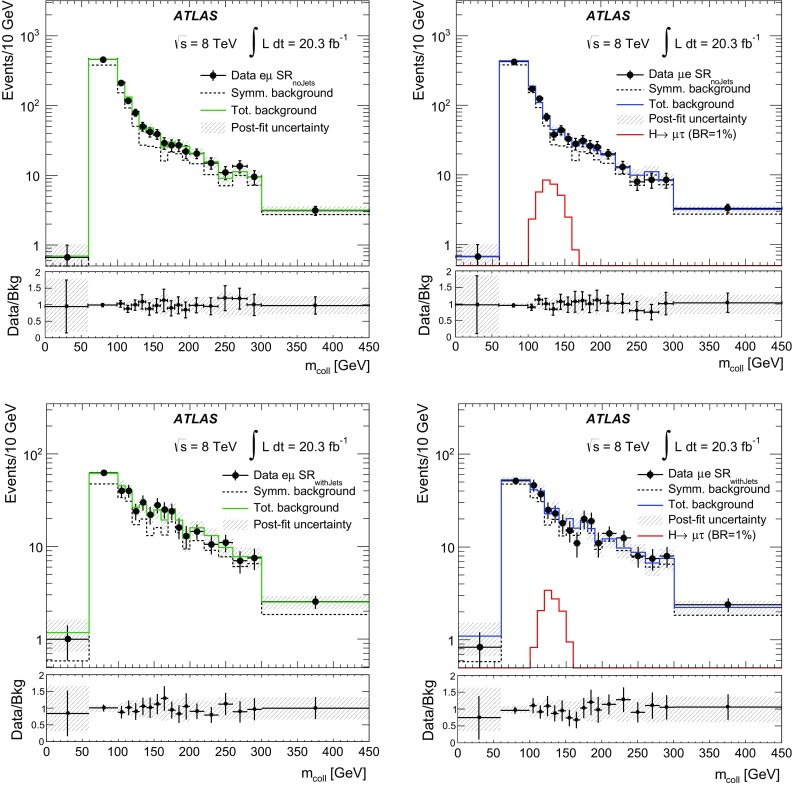
Collinear mass distributions in the $\tau _{\mathrm {lep}} {}$ channel: background estimate compared to the events observed in the data in the $\mathrm {SR_{noJets}}$ (*top*) and $\mathrm {SR_{withJets}}$ (*bottom*). *Left* $e\mu$ channel. *Right* $\mu e$ channel. In these plots, events from the three $f\left( p_{\mathrm {T}}^{\ell {}_{2}}\right)$ bins are combined, although the fit parameters are different in each $f\left( p_{\mathrm {T}}^{\ell {}_{2}}\right)$ bin. The signal expected for a $\text {Br}(H \rightarrow \mu {}\tau {}{})=1\%$ is shown in the $\mu e$ channel

Figure 4 compares the observed data to the yields expected from the symmetry-based statistical model. The comparison, combining the different $p_{\mathrm {T}}^{\ell {}_{2}}$ bins, shows the symmetric component of the background ($b_{ij}$) as a dashed line, and the total background estimation including the contribution from events containing misidentified and non-prompt leptons as a full line. As can be seen, the background estimation is in good agreement with the data over the full mass range. Table 5 summarises the fit results in the data in $\mathrm {SR_{noJets}}$ and $\mathrm {SR_{withJets}}$: the fitted $f\left( p_{\mathrm {T}}^{\ell {}_{2}}\right)$ scale factors, the symmetric background component ($\sum \limits _{i}^{N_{m_\mathrm{coll}}}b_{ij}$) in each $p_{\mathrm {T}}^{\ell {}_{2}}$ bin, and the non-prompt estimate in the $\mu e$ and the $e\mu$ channels. The excellent level of agreement between the fitted number of events and the observed number is due to the many unconstrained parameters in the fit.

**Table 5:** A summary of the fit results in the $\tau _{\mathrm {lep}} {}$ channel. The values of the fit parameters $f\left( p_{\mathrm {T}}^{\ell {}_{2}}\right)$, which account for the ratio of the $e\mu$ and $\mu e$ event reconstruction efficiencies described in Sect. 5.2, are obtained from a background-only fit, and reported for each signal region and for each $p_{\mathrm {T}}^{\ell {}_{2}}$ bin. The expected and observed yields correspond to the number of events used in the fit, representing the 0–300 GeV $m_\mathrm{coll}$ range shown in Fig. 4. The quoted uncertainties in the expected yields represent the statistical (first) and systematic (second) uncertainties, respectively. The post-fit values of systematic uncertainties are provided for the background predictions. The signal predictions are given for $\text {Br}(H \rightarrow {}e\tau {}{})=1\%$ in the $e\mu$ sample and for $\text {Br}(H \rightarrow \mu {}\tau {}{})=1\%$ in the $\mu e$ sample

$p_{\mathrm {T}}^{\ell {}_{2}}$ bin (GeV)	$f\left( p_{\mathrm {T}}^{\ell {}_{2}}\right)$		LFV Signal, $\mathrm{Br}=1\%$	Total backg.	Observed
$$\mathrm {SR_{noJets}}$$					
12–20	$1.11 \pm 0.06$	$e\mu$	$14.9 \pm 0.4 \pm 2.7$	$1219 \pm 24 \pm 27$	1212
		$\mu e$	$10.7 \pm 0.4 \pm 2.3$	$1033 \pm 25 \pm 20$	1035
20–30	$1.07 \pm 0.08$	$e\mu$	$15.1 \pm 0.4 \pm 2.7$	$998 \pm 22 \pm 25$	995
		$\mu e$	$12.4 \pm 0.4 \pm 2.2$	$950 \pm 23 \pm 21$	950
$\ge 30$	$1.01 \pm 0.07$	$e\mu$	$12.5 \pm 0.4 \pm 2.2$	$455 \pm 17 \pm 16$	452
		$\mu e$	$11.4 \pm 0.4 \pm 2.0$	$458 \pm 16 \pm 14$	457
$$\mathrm {SR_{withJets}}$$					
12–20	$1.07 \pm 0.10$	$e\mu$	$5.9 \pm 0.3 \pm 1.1$	$222 \pm 10 \pm 11$	220
		$\mu e$	$3.9 \pm 0.2 \pm 0.9$	$181 \pm 10 \pm 9$	182
20–30	$1.24 \pm 0.16$	$e\mu$	$5.4 \pm 0.2 \pm 1.1$	$187 \pm 9 \pm 11$	187
		$\mu e$	$4.5 \pm 0.2 \pm 0.9$	$161 \pm 9 \pm 9$	161
$\ge 30$	$1.13 \pm 0.10$	$e\mu$	$5.5 \pm 0.2 \pm 1.0$	$251 \pm 11 \pm 12$	250
		$\mu e$	$4.9 \pm 0.2 \pm 0.9$	$229 \pm 11 \pm 11$	229

The expected and observed 95$\%$ CL upper limits on branching ratios as well as their best fit values are calculated using the statistical model described in Sect. 5.4. Table 6 presents a summary of results for the individual categories and their combination can be found in Table 6 for both the $H \rightarrow {}e\tau {}$ and $H \rightarrow \mu {}\tau {}$ hypotheses.

## Combined results of the search for LFV $H\rightarrow e\tau /\mu \tau$ decays

**Fig. 5:**
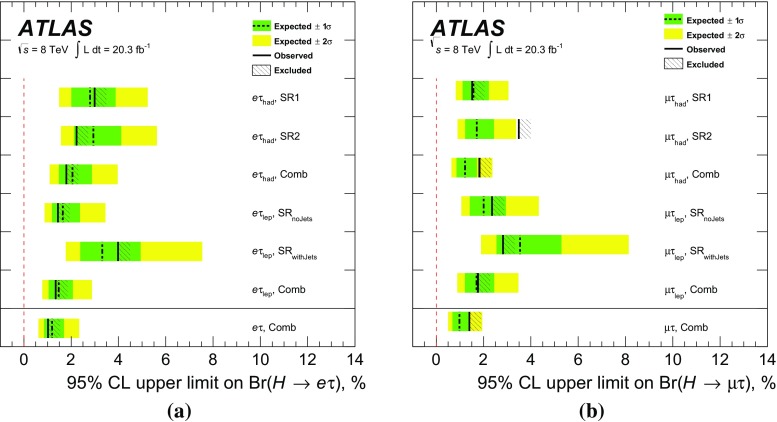
Upper limits on LFV decays of the Higgs boson in the $H \rightarrow {}e\tau {}$ hypothesis (*left*) and $H \rightarrow \mu {}\tau {}$ hypothesis (*right*). The limits are computed under the assumption that either Br($H\rightarrow {}\mu {}\tau {}$)=0 or Br($H\rightarrow {}e\tau {}$)=0. The $\mu \tau _{\mathrm {had}}$ channel is from Ref. [22]

The results of the individual searches for the LFV $H\rightarrow e\tau$ and $H\rightarrow \mu \tau$ decays in the $\tau _{\mathrm {had}}$ (including the result from Ref. [22]) and $\tau _{\mathrm {lep}}$ channels presented in Sects. 4.4 and 5.6 are statistically combined. The two channels use different background estimation techniques, leading to uncorrelated systematic uncertainties in the background predictions. The systematic uncertainties for the LFV signal are treated as 100$\%$ correlated between the two channels. Table 6 presents a summary of results for the expected and observed $95\%$ CL upper limits and the best fit values for the branching ratios for the individual categories and their combination. There is no indication of a signal in the search for the LFV $H\rightarrow e\tau$ decays. The combined observed, and the median expected, 95$\%$ CL upper limits on Br($H\rightarrow e\tau$) for a Higgs boson with $m_{H} = 125$ GeV are 1.04$\%$ and $1.21^{+0.49}_{-0.34}$ $\%$, respectively. A small $\sim$1$\sigma$ excess of data over the predicted background is observed in the search for the LFV $H\rightarrow \mu \tau$ decays. It is mostly driven by a 1.3$\sigma$ excess in the earlier search in the $\mu \tau _{\mathrm {had}}$ channel [22]. This corresponds to a best fit value for the branching ratio of Br($H\rightarrow \mu \tau$) = ($0.53 \pm 0.51$)$\%$. In the absence of any significant signal, an upper limit on the LFV branching ratio Br($H\rightarrow \mu \tau$) for a Higgs boson with $m_\mathrm {H} = 125$ GeV is set. The corresponding observed, and the median expected, 95$\%$ CL upper limits are 1.43$\%$ and $1.01^{+0.40}_{-0.29}$ $\%$, respectively. The upper limits on the LFV decays of the Higgs boson are summarised in Fig. 5.

**Table 6:** Results of the search for the LFV $H\rightarrow e\tau$ and $H\rightarrow \mu \tau$ decays. The limits are computed under the assumption that either Br($H\rightarrow {}\mu {}\tau {}$)=0 or Br($H\rightarrow {}e\tau {}$)=0. The expected and observed $95\%$ confidence level (CL) upper limits and the best fit values for the branching ratios for the individual categories and their combination. The $\mu \tau _{\mathrm {had}}$ channel is from Ref. [22]

Channel	Category	Expected limit ($\%$)	Observed limit ($\%$)	Best fit Br (%)
	SR1	$2.81^{+1.06}_{-0.79}$	3.0	$0.33^{+1.48}_{-1.59}$
$H\rightarrow e \tau _{\mathrm {had}}$	SR2	$2.95^{+1.16}_{-0.82}$	2.24	$-1.33^{+1.56}_{-1.80}$
	Combined	$2.07^{+0.82}_{-0.58}$	1.81	$-0.47^{+1.08}_{-1.18}$
	$\mathrm {SR_{noJets}}$	$1.66^{+0.72}_{-0.46}$	1.45	$-0.45^{+0.89}_{-0.97}$
$H\rightarrow e \tau _{\mathrm {lep}}$	$\mathrm {SR_{withJets}}$	$3.33^{+1.60}_{-0.93}$	3.99	$0.74^{+1.59}_{-1.62}$
	Combined	$1.48^{+0.60}_{-0.42}$	1.36	$-0.26^{+0.79}_{-0.82}$
$H\rightarrow e\tau$	Combined	$1.21^{+0.49}_{-0.34}$	1.04	$-0.34^{+0.64}_{-0.66}$
	SR1	$1.60^{+0.64}_{-0.45}$	1.55	$-0.07^{+0.81}_{-0.86}$
$H\rightarrow \mu \tau _{\mathrm {had}}$	SR2	$1.75^{+0.71}_{-0.49}$	3.51	$1.94^{+0.92}_{-0.89}$
	Combined	$1.24^{+0.50}_{-0.35}$	1.85	$0.77^{+0.62}_{-0.62}$
	$\mathrm {SR_{noJets}}$	$2.03^{+0.93}_{-0.57}$	2.38	$0.31^{+1.06}_{-0.99}$
$H\rightarrow \mu \tau _{\mathrm {lep}}$	$\mathrm {SR_{withJets}}$	$3.57^{+1.74}_{-1.00}$	2.85	$-1.03^{+1.66}_{-1.82}$
	Combined	$$1.73^{+0.74}_{-0.49}$$	1.79	$$ 0.03^{+0.88}_{-0.86}$$
$H\rightarrow \mu \tau$	Combined	$1.01^{+0.40}_{-0.29}$	1.43	$0.53^{+0.51}_{-0.51}$

## Search for $Z\rightarrow \mu \tau$ using the $\tau _{\mathrm {had}}$ channel

The search for $Z\rightarrow \mu \tau$ events is based on $\mu \tau _{\mathrm {had}}$ final state and utilises the same strategy as the $H\rightarrow \mu \tau$ analysis documented in Ref. [22], and applied to the $H\rightarrow e \tau _{\mathrm {had}}$ search described above. The final state is characterised by the presence of an energetic muon and a $\tau _{\mathrm {had}}$ of opposite charge and the presence of moderate $E_\mathrm {T}^\mathrm {miss} {},$ aligned with the $\tau _{\mathrm {had}}$ direction. The typical transverse momenta of the muon and of the $\tau _{\mathrm {had}}$ are somewhat softer than those expected in Higgs boson LFV decay, due to the lower mass of the *Z* boson. The main backgrounds are the same as those observed in $H\rightarrow \mu \tau _{\mathrm {had}}$ analyses, namely: $Z\rightarrow \tau \tau$, *W*+jets, multi-jet, $H\rightarrow \tau \tau$, diboson and top backgrounds. The $m_{\mu \tau }^{\mathrm {MMC}}$ variable is used to extract the signal using the same fit procedure and estimation of systematic uncertainties as for the $H\rightarrow \mu \tau _{\mathrm {had}}$ search. The corresponding Higgs boson LFV contribution is assumed to be negligible.

The $Z\rightarrow \mu \tau$ analysis differs from the $H\rightarrow \mu \tau _{\mathrm {had}}$ one as follows:

- The signal and control regions are defined in the same way as in the $H\rightarrow \mu \tau _{\mathrm {had}}$ analysis, but the cut values are lowered to match the kinematics of *Z* boson decay products. The exact definition is given in Table 7.
- The LFV $H\rightarrow \mu \tau _{\mathrm {had}}$ signal sample is replaced with a LFV $Z\rightarrow \mu \tau$ signal sample.
- The shape correction for *W*+jets in SR1 is obtained from the $m_{\mu \tau }^{\mathrm {MMC}}>110$ GeV sideband in SR1.
- Due to larger *W*+jets contribution in SR1 and SR2, the shape corrections for the *W*+jets samples are calculated using a three-dimensional binning scheme in $p_{\text {T}} ( \tau _{\mathrm {had}} )$, $|\eta (\mu )-\eta ( \tau _{\mathrm {had}} )|$ and $N_{\mathrm {jet}}$.
- The *W*+jets extrapolation uncertainty, which accounts for the difference between the *W*+jets ALPGEN PYTHIA and HERWIG samples, is also included as a shape uncertainty.

**Fig. 6:**
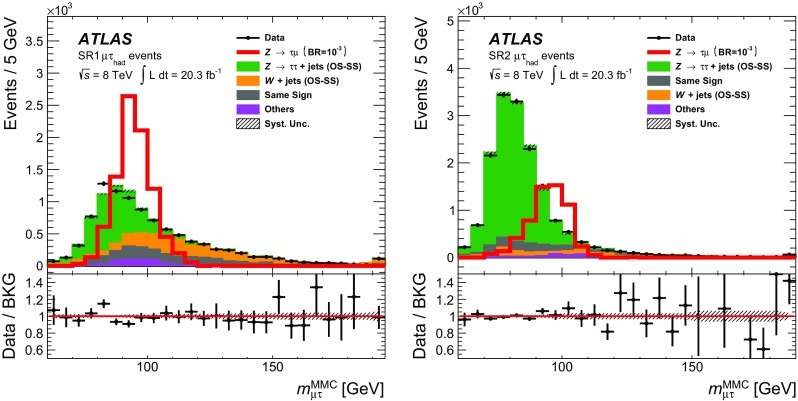
Distributions of the mass reconstructed by the Missing Mass Calculator, $m_{\mu \tau }^{\mathrm {MMC}}$, in $Z\rightarrow \mu \tau$ SR1 (*left*) and SR2 (*right*). The background distributions are determined in a global fit. The signal distributions are scaled to a branching ratio of Br($Z\rightarrow \mu \tau$) = $10^{-3}$ to make them visible. The *bottom panel* of each subfigure shows the ratio of the observed data to the estimated background. The *hatched band* for the ratio illustrates post-fit systematic uncertainties in the background prediction. The statistical uncertainties for data and background predictions are added in quadrature for the ratios. The last bin of the distribution contains events with $m_{\mu \tau }^{\mathrm {MMC}}>200$ GeV

The numbers of observed events and background in each of the regions are given in Table 8. The efficiencies for simulated $Z\rightarrow \mu \tau$ signal events to pass the SR1 and SR2 selections are 1.2 and 0.8%, respectively. Figure 6 shows the $m_{\mu \tau }^{\mathrm {MMC}}$ distribution for data and predicted background in each of the signal regions. The discrepancy observed in the $m_{\mu \tau }^{\mathrm {MMC}}$ range 80–100 GeV of SR1 was studied carefully. All the other SR1 distributions, including lepton momenta, transverse masses, and missing transverse momentum, are in excellent agreement with the predictions, and the background shapes are constrained in the control regions as well as in SR2. This discrepancy is hence attributed to a statistical fluctuation.

**Table 7:** Summary of the $Z\rightarrow \mu \tau _{\mathrm {had}}$ event selection criteria used to define the signal and control regions (see text)

Cut	SR1	SR2	WCR	TCR
$p_{\text {T}} (\mu )$	>30 GeV	>30 GeV	>30 GeV	>30 GeV
$p_{\text {T}} ( \tau _{\mathrm {had}} )$	>30 GeV	>30 GeV	>30 GeV	>30 GeV
$|\eta (\mu )-\eta ( \tau _{\mathrm {had}} )|$	<2	<2	<2	<2
$m_{\mathrm {T}}^{\mu ,E_{\text {T}}^{\text {miss}}}$	>30 and <75 GeV	<30 GeV	>60 GeV	–
$m_{\mathrm {T}}^{ \tau _{\mathrm {had}} ,E_{\text {T}}^{\text {miss}}}$	<20 GeV	<45 GeV	>40 GeV	–
$N_{\mathrm {jet}}$	–	–	–	>1
$N_{b-\mathrm {jet}}$	0	0	0	>0

**Table 8:** Data yields, signal and post-fit OS–SS background predictions (see Eq. (1)) for the $Z\rightarrow \mu \tau _{\mathrm {had}}$ 80 GeV$<m_{\mu \tau }^{\mathrm {MMC}}<$115 GeV region. The signal predictions are given assuming Br($Z\rightarrow \mu \tau$) = $10^{-5}$. The background predictions are obtained from the combined fit to SR1, SR2, WCR and TCR. To calculate these quantities for SR1 and SR2, the signal strengths are decorrelated in the signal regions and set to zero in the control regions. The post-fit values of systematic uncertainties are provided for the background predictions. For the total background, all correlations between various sources of systematic uncertainties and backgrounds are taken into account. The quoted uncertainties represent the statistical (first) and systematic (second) uncertainties, respectively

	SR1			SR2		
Signal	86	±2	±22	56	±2	±18
$Z\rightarrow \tau \tau$	3260	±30	±60	7060	±40	±150
*W*+jets	1350	±70	±110	590	±50	±70
Same-Sign events	1110	±40	±100	930	±30	±90
$VV+Z\rightarrow \mu \mu$	410	±60	±50	240	±60	±60
$H\rightarrow \tau \tau$	25.1	± 0.5	±3.0	41	± 1	±5
Top	22	±4	±4	15	±4	±4
Total background	6170	±100	±100	8880	±100	±140
Data	6134			8982		

No excess of data is observed and the CL$_s$ limit-setting technique is used to calculate the observed and expected limits on the branching ratio for $Z\rightarrow \mu \tau$  decays. The observed 95 % CL limit on Br($Z\rightarrow \mu \tau$) is $1.7 \times 10^{-5}$, which is lower than the expected upper limit of Br($Z\rightarrow \mu \tau$)$= 2.6 \times 10^{-5}$, but still within the $2 \sigma$ band. This corresponds to a best fit value for the branching ratio Br($Z\rightarrow \mu \tau$)$= -1.6_{-1.4}^{+1.3} \times 10^{-5}$. The results for the different signal regions are summarised in Table 9.

**Table 9:** The expected and observed 95% CL exclusion limits as well as the best fit values for the branching ratio of $\text {Br}(Z\rightarrow \mu \tau )[10^{-5}]\,$ are shown for SR1, SR2 and the combined fit. To calculate these quantities for SR1 and SR2, the signal strengths are decorrelated in the signal regions and set to zero in the control regions

$\text {Br}(Z\rightarrow \mu \tau )\, (10^{-5})$	SR1	SR2	Combined
Expected limit	$2.6_{-0.7}^{+1.1}$	$6.4^{-1.8}_{+2.8}$	$2.6_{-0.7}^{+1.1}$
Observed limit	1.5	7.9	1.7
Best fit	$-2.1^{+1.2}_{-1.3}$	$2.6^{+2.9}_{-2.6}$	$-1.6_{-1.4}^{+1.3}$

## Summary

Searches for lepton-flavour-violating decays of the *Z* and Higgs bosons are performed using a data sample of proton–proton collisions recorded by the ATLAS detector at the LHC corresponding to an integrated luminosity of 20.3 $\mathrm{fb}^{-1}$ at $\sqrt{s}=8$ TeV. Three LFV decays are considered: $H\rightarrow e\tau$, $H\rightarrow \mu \tau$, and $Z\rightarrow \mu \tau$. The search for the Higgs boson decays is performed in the final states where the $\tau$-lepton decays either to hadrons or to leptons (electron or muon). The search for the *Z* boson decays is performed in the final state with the $\tau$-lepton decaying into hadrons. No significant excess is observed, and upper limits on the LFV branching ratios are set. The observed and the median expected 95$\%$ CL upper limits on Br($H\rightarrow e\tau$) are $1.04$ $\%$ and $1.21^{+0.49}_{-0.34}$ $\%$, respectively. This direct search for the $H\rightarrow e\tau$ decays places significantly more stringent constraints on Br($H\rightarrow e\tau$) than earlier indirect estimates. In the search for the $H\rightarrow \mu \tau$ decays, the observed and the median expected 95$\%$ CL upper limits on Br($H\rightarrow \mu \tau$) are $1.43$ $\%$ and $1.01^{+0.40}_{-0.29}$ $\%$, respectively. A small deficit of data compared to the predicted background is observed in the search for the LFV $Z\rightarrow \mu \tau$ decays. The observed and the median expected 95$\%$ CL upper limits on Br($Z\rightarrow \mu \tau$) are $1.69\times 10^{-5}$ and $2.58\times 10^{-5}$, respectively.

## Footnotes

1 ATLAS uses a right-handed coordinate system with its origin at the nominal interaction point (IP) in the centre of the detector and the *z*-axis along the beam pipe. The *x*-axis points from the IP to the centre of the LHC ring, and the *y*-axis points upward. Cylindrical coordinates $(r,\phi )$ are used in the transverse plane, $\phi$ being the azimuthal angle around the beam pipe. The pseudorapidity is defined in terms of the polar angle $\theta$ as $\eta =-\ln \tan (\theta /2)$. The transverse momentum and the transverse energy are defined as $p_{\text {T}} =p\times \sin (\theta )$ and $E_{\text {T}} =E\times \sin (\theta )$, respectively. The distance $\Delta {}R$ in $\eta$-$\phi$ space is defined as $\Delta R~=~\sqrt{(\Delta \eta )^{2} + (\Delta \phi )^{2}}$.

2
$m_{\mathrm {T}}^{\ell ,E_{\text {T}}^{\text {miss}}}=\sqrt{2p_{\text {T}} ^{\ell }E_{\text {T}}^{\text {miss}} (1-\cos {\Delta \phi })}$, where $\ell =e$, $\tau _{\mathrm {had}}$ and $\Delta \phi$ is the azimuthal separation between the directions of the lepton (*e* or $\tau _{\mathrm {had}}$) and $E_{\text {T}}^{\text {miss}}$ vectors.

3 The $r_{\mathrm {QCD}}\cdot N_{\mathrm {SS}}^{\mathrm {bkg}{\text {-}}i}$ correction in the *add-on* term is needed because same-sign data events include multi-jet as well as electroweak events ($Z\rightarrow \tau \tau$, $Z\rightarrow ee$, *W*+jets, *VV*, $H\rightarrow \tau \tau$ and events with *t*-quarks) and their contributions cannot be separated.

4 The same extrapolation uncertainty is assumed for $t\bar{t}$ and single-top backgrounds.

5 The effect of the mass difference between electrons and muons is negligible for the processes involved.

## References

[1] J. Bjorken, S. Weinberg, A mechanism for nonconservation of muon number. Phys. Rev. Lett. **38**, 622 (1977). doi:10.1103/PhysRevLett.38.622

[2] Diaz-Cruz JL, Toscano J (2000). Lepton flavor violating decays of Higgs bosons beyond the standard model. Phys. Rev. D.

[3] M. Arana-Catania, E. Arganda, M.J. Herrero, Non-decoupling SUSY in LFV Higgs decays: a window to new physics at the LHC. JHEP **1309**, 160 (2013). doi:10.1007/JHEP09(2013)160. arXiv:1304.3371 [hep-ph].** (Erratum: JHEP 1510, 192 (2015). **http://dx.doi.org/. doi:10.1007/JHEP10(2015)192. arXiv:1304.3371** [hep-ph])**

[4] Arhrib A, Cheng Y, Kong OC (2013). Comprehensive analysis on lepton flavor violating Higgs boson to $$\mu ^\mp \tau ^\pm $$ decay in supersymmetry without $$R$$ parity. Phys. Rev. D.

[5] Agashe K, Contino R (2009). Composite Higgs-mediated FCNC. Phys. Rev. D.

[6] Azatov A, Toharia M, Zhu L (2009). Higgs mediated FCNC’s in warped extra dimensions. Phys. Rev. D.

[7] Ishimori H (2010). Non-abelian discrete symmetries in particle physics. Prog. Theor. Phys. Suppl..

[8] Perez G, Randall L (2009). Natural neutrino masses and mixings from warped geometry. JHEP.

[9] Blanke M, Buras AJ, Duling B, Gori S, Weiler A (2009). $$\Delta F=2$$ observables and fine-tuning in a warped extra dimension with custodial protection. JHEP.

[10] Giudice GF, Lebedev O (2008). Higgs-dependent Yukawa couplings. Phys. Lett. B.

[11] Aguilar-Saavedra J (2009). A minimal set of top-Higgs anomalous couplings. Nucl. Phys. B.

[12] Albrecht ME, Blanke M, Buras AJ, Duling B, Gemmler K (2009). Electroweak and flavour structure of a warped extra dimension with custodial protection. JHEP.

[13] Goudelis A, Lebedev O, Park J-H (2012). Higgs-induced lepton flavor violation. Phys. Lett. B.

[14] McKeen D, Pospelov M, Ritz A (2012). Modified Higgs branching ratios versus CP and lepton flavor violation. Phys. Rev. D.

[15] Crivellin A, D’Ambrosio G, Heeck J (2015). Addressing the LHC flavor anomalies with horizontal gauge symmetries. Phys. Rev. D.

[16] Crivellin A, D’Ambrosio G, Heeck J (2015). Explaining $$h\rightarrow \mu ^\pm \tau ^\mp $$, $$B\rightarrow K^* \mu ^+\mu ^-$$ and $$B\rightarrow K \mu ^+\mu ^-/B\rightarrow K e^+e^-$$ in a two-Higgs-doublet model with gauged $$L_\mu -L_\tau $$. Phys. Rev. Lett..

[17] Illana JI, Riemann T (2001). Charged lepton flavor violation from massive neutrinos in Z decays. Phys. Rev. D.

[18] Kuo T-K, Nakagawa N (1985). Lepton flavor violating decays of $$Z^0$$ and $$\tau $$. Phys. Rev. D.

[19] Gabbiani F, Kim JH, Masiero A (1988). $$Z^0 \rightarrow b \bar{s}$$ and $$Z^0 \rightarrow \tau \bar{\mu }$$ in SUSY: are they observable?. Phys. Lett. B.

[20] Olive K (2014). Review of particle physics. Chin. Phys. C.

[21] CMS Collaboration, Search for lepton-flavour-violating decays of the Higgs boson. Phys. Lett. B **749**, 337–362 (2015). 10.1016/j.physletb.2015.07.053. arXiv:1502.07400 [hep-ex]

[22] ATLAS Collaboration, Search for lepton-flavour-violating $$H\rightarrow \mu \tau $$ decays of the Higgs boson with the ATLAS detector. JHEP **1511**, 211 (2015). doi:10.1007/JHEP11(2015)211. arXiv:1508.03372 [hep-ex]

[23] M.E.G. Collaboration, J. Adam et al., New constraint on the existence of the $$\mu ^+ \rightarrow e^+\gamma $$ decay. Phys. Rev. Lett. **110**, 201801 (2013). doi:10.1103/PhysRevLett.110.201801. arXiv:1303.0754 [hep-ex] 10.1103/PhysRevLett.110.201801 25167396

[24] Harnik R, Kopp J, Zupan J (2013). Flavor violating Higgs decays. JHEP.

[25] Blankenburg G, Ellis J, Isidori G (2012). Flavour-changing decays of a 125 GeV Higgs-like particle. Phys. Lett. B.

[26] Collaboration OPAL, Akers R (1995). A search for lepton flavor violating $$Z^0$$ decays. Z. Phys. C.

[27] DELPHI Collaboration, P. Abreu et al., Search for lepton flavor number violating $$Z^0$$ decays. Z. Phys. C **73**, 243–251 (1997). doi:10.1007/s002880050313

[28] ATLAS Collaboration, Search for the lepton flavor violating decay $$Z\rightarrow e \mu $$ in pp collisions at $$\sqrt{s}$$ 8 TeV with the ATLAS detector. Phys. Rev. D **90**, 072010 (2014). doi:10.1103/PhysRevD.90.072010. arXiv:1408.5774 [hep-ex]

[29] Bressler S, Dery A, Efrati A (2014). Asymmetric lepton-flavor violating Higgs boson decays. Phys. Rev. D.

[30] ATLAS Collaboration, The ATLAS experiment at the CERN Large Hadron Collider. JINST **3**, S08003 (2008). doi:10.1088/1748-0221/3/08/S08003

[31] ATLAS Collaboration, Performance of pile-up mitigation techniques for jets in $$pp$$ collisions at $$\sqrt{s} = 8$$ TeV using the ATLAS detector. Eur. Phys. J. C 76, 581 (2016) doi:10.1140/epjc/s10052-016-4395-z 10.1140/epjc/s10052-016-4395-z PMC5335592 28316490

[32] ATLAS Collaboration, Measurement of the muon reconstruction performance of the ATLAS detector using 2011 and 2012 LHC proton–proton collision data. Eur. Phys. J. C **74**, 3130 (2014). doi:10.1140/epjc/s10052-014-3130-x. arXiv:1407.3935 [hep-ex] 10.1140/epjc/s10052-014-3130-x PMC4371046 25814875

[33] ATLAS Collaboration, Electron reconstruction and identification efficiency measurements with the ATLAS detector using the 2011 LHC proton–proton collision data. Eur. Phys. J. C **74**, 2941 (2014). DOI:10.1140/epjc/s10052-014-2941-0. arXiv:1404.2240 [hep-ex] 10.1140/epjc/s10052-014-2941-0 PMC4371047 25814900

[34] ATLAS Collaboration, Identification and energy calibration of hadronically decaying tau leptons with the ATLAS experiment in $$pp$$ collisions at $$\sqrt{s}$$ =8 TeV. Eur. Phys. J. C **75**, 303 (2015). doi:10.1140/epjc/s10052-015-3500-z. arXiv:1412.7086 [hep-ex] 10.1140/epjc/s10052-015-3500-z PMC4498687 26190938

[35] ATLAS Collaboration, Evidence for the Higgs-boson Yukawa coupling to tau leptons with the ATLAS detector. JHEP **1504**, 117 (2015). doi:10.1007/JHEP04(2015)117. arXiv:1501.04943 [hep-ex]

[36] Cacciari M, Salam GP, Soyez G (2008). The anti-kt jet clustering algorithm. JHEP.

[37] ATLAS Collaboration, Single hadron response measurement and calorimeter jet energy scale uncertainty with the ATLAS detector at the LHC. Eur. Phys. J. C **73**, 2305 (2013). 10.1140/epjc/s10052-013-2305-1. arXiv:1203.1302 [hep-ex] 10.1140/epjc/s10052-016-4580-0 PMC5312118 28260979

[38] ATLAS Collaboration, Calibration of the performance of $$b$$-tagging for $$c$$ and light-flavour jets in the 2012 ATLAS data. ATLAS-CONF-2014-046 (2014). http://cdsweb.cern.ch/record/1741020

[39] ATLAS Collaboration, Performance of missing transverse momentum reconstruction in proton–proton collisions at 7 TeV with ATLAS. Eur. Phys. J. C **72**, 1844 (2012). doi:10.1140/epjc/s10052-011-1844-6. arXiv:1108.5602 [hep-ex]

[40] ATLAS Collaboration, Modelling $$Z\rightarrow \tau \tau $$ processes in ATLAS with $$\tau $$-embedded $$Z\rightarrow \mu \mu $$ data. JINST **10**(09), P09018 (2015). doi:10.1088/1748-0221/10/09/P09018. arXiv:1506.05623 [hep-ex]

[41] Mangano ML, Moretti M, Piccinini F, Pittau R, Polosa AD (2003). ALPGEN, a generator for hard multiparton processes in hadronic collisions. JHEP.

[42] Sjöstrand T, Mrenna S, Skands PZ, Brief A (2008). Introduction to PYTHIA 8.1. Comput. Phys. Commun..

[43] Nason P (2004). A new method for combining NLO QCD with shower Monte Carlo algorithms. JHEP.

[44] Frixione S, Nason P, Oleari C (2007). Matching NLO QCD computations with Parton shower simulations: the POWHEG method. JHEP.

[45] Alioli S, Nason P, Oleari C, Re E (2010). A general framework for implementing NLO calculations in shower Monte Carlo programs: the POWHEG BOX. JHEP.

[46] Kersevan BP, Richter-Was E (2013). The Monte Carlo event generator AcerMC versions 2.0 to 3.8 with interfaces to PYTHIA 6.4, HERWIG 6.5 and ARIADNE 4.1. Comput. Phys. Commun..

[47] Corcella G (2001). HERWIG 6: an event generator for hadron emission reactions with interfering gluons (including supersymmetric processes). JHEP.

[48] Alioli S, Nason P, Oleari C, Re E (2009). NLO Higgs boson production via gluon fusion matched with shower in POWHEG. JHEP.

[49] Anastasiou C, Melnikov K (2002). Higgs boson production at hadron colliders in NNLO QCD. Nucl. Phys. B.

[50] Ravindran V, Smith J, van Neerven WL (2003). NNLO corrections to the total cross-section for Higgs boson production in hadron hadron collisions. Nucl. Phys. B.

[51] Bolzoni P, Maltoni F, Moch S-O, Zaro M (2010). Higgs production via vector-boson fusion at NNLO in QCD. Phys. Rev. Lett..

[52] Lange DJ (2001). The EvtGen particle decay simulation package. Nucl. Instrum. Meth. A.

[53] S. Jadach, Z. Wa̧s, R. Decker, J. H. Kuhn, The tau decay library TAUOLA: version 2.4. Comput. Phys. Commun. **76**, 361–380 (1993). doi:10.1016/0010-4655(93)90061-G

[54] ATLAS Collaboration, The ATLAS simulation infrastructure. Eur. Phys. J. C **70**, 823 (2010). doi:10.1140/epjc/s10052-010-1429-9. arXiv:1005.4568 [physics.ins-det]

[55] GEANT4 Collaboration, S. Agostinelli et al., GEANT4: a simulation toolkit. Nucl. Instrum. Meth. A **506**, 250 (2003). doi:10.1016/S0168-9002(03)01368-8

[56] A. Elagin, P. Murat, A. Pranko, A. Safonov, A new mass reconstruction technique for resonances decaying to di-tau. Nucl. Instrum. Meth. A **654**, 481 (2011). doi:10.1016/j.nima.2011.07.009. arXiv:1012.4686 [hep-ex]

[57] Ellis RK, Hinchliffe I, Soldate M, van der Bij JJ (1988). Higgs decay to $$\tau ^{+}\tau ^{-}$$: a possible signature of intermediate mass higgs bosons at the SSC. Nucl. Phys. B.

[58] ATLAS Collaboration, Search for the Standard Model Higgs boson in the $$H\rightarrow \tau ^{+}\tau ^{-}$$ decay mode in $$\sqrt{s}=7$$ TeV $$pp$$ collisions with ATLAS. JHEP **1209**, 070 (2012). doi:10.1007/JHEP09(2012)070. arXiv:1206.5971 [hep-ex]

[59] ATLAS Collaboration, Measurement of the $$Z\rightarrow \tau \tau $$ cross section with the ATLAS detector. Phys. Rev. D **84**, 112006 (2011). doi:10.1103/PhysRevD.84.112006. arXiv:1108.2016 [hep-ex]

[60] Frixione S, Webber BR (2002). Matching NLO QCD computations and parton shower simulations. JHEP.

[61] Campbell JM, Ellis RK, Williams C (2011). Vector boson pair production at the LHC. JHEP.

[62] ATLAS Collaboration, Jet energy measurement and its systematic uncertainty in proton–proton collisions at $$\sqrt{s}=7$$ TeV with the ATLAS detector. Eur. Phys. J. C **75**, 17 (2015). doi:10.1140/epjc/s10052-014-3190-y. arXiv:1406.0076 [hep-ex] 10.1140/epjc/s10052-014-3190-y PMC4684939 26709345

[63] ATLAS Collaboration, Jet energy resolution in proton–proton collisions at $$\sqrt{s}=7$$ TeV recorded in 2010 with the ATLAS detector. Eur. Phys. J. C **73**, 2306 (2013). doi:10.1140/epjc/s10052-013-2306-0. arXiv:1210.6210 [hep-ex] 10.1140/epjc/s10052-013-2306-0 PMC4371084 25814854

[64] ATLAS Collaboration, Improved luminosity determination in pp collisions at sqrt(s) = 7 TeV using the ATLAS detector at the LHC. Eur. Phys. J. C **73**, 2518 (2013). doi:10.1140/epjc/s10052-013-2518-3. arXiv:1302.4393 [hep-ex] 10.1140/epjc/s10052-013-2518-3 PMC4370906 25814867

[65] LHC Higgs Cross Section Working Group Collaboration, S. Dittmaier et al., Handbook of LHC Higgs Cross Sections: 1. Inclusive Observables. arXiv:1101.0593 [hep-ph]

[66] Read AL (2002). Presentation of search results: the CL(s) technique. J. Phys. G.

[67] G. Cowan, K. Cranmer, E. Gross, O. Vitells, Asymptotic formulae for likelihood-based tests of new physics. Eur. Phys. J. C **71**, 1554 (2011). doi:10.1140/epjc/s10052-011-1554-0. arXiv:1007.1727 [physics.data-an]. (Erratum: Eur. Phys. J. C 73, 2501 (2013). doi:10.1140/epjc/s10052-013-2501-z. arXiv:1007.1727 [physics.data-an])

[68] ATLAS Collaboration, Search for direct production of charginos, neutralinos and sleptons in final states with two leptons and missing transverse momentum in $$pp$$ collisions at $$\sqrt{s} =$$ 8 TeV with the ATLAS detector. JHEP **1405**, 071 (2014). doi:10.1007/JHEP05(2014)071. arXiv:1403.5294 [hep-ex]

[69] O. Behnke, K. Kröninger, T. Schörner-Sadenius, G. Schott, eds., Data Analysis in High Energy Physics. Wiley-VCH, Weinheim (2013). doi:10.1002/9783527653416

[70] ATLAS Collaboration, ATLAS computing acknowledgements 2016–2017. ATL-GEN-PUB-2016-002 (2016). https://cds.cern.ch/record/2202407

